# A missense mutation in the RSRSP stretch of *Rbm20* causes dilated cardiomyopathy and atrial fibrillation in mice

**DOI:** 10.1038/s41598-020-74800-8

**Published:** 2020-10-27

**Authors:** Kensuke Ihara, Tetsuo Sasano, Yuichi Hiraoka, Marina Togo-Ohno, Yurie Soejima, Motoji Sawabe, Megumi Tsuchiya, Hidesato Ogawa, Tetsushi Furukawa, Hidehito Kuroyanagi

**Affiliations:** 1 Department of Bio-informational Pharmacology, Medical Research Institute, Tokyo Medical and Dental University (TMDU), Tokyo, 113-8510 Japan; 2 Department of Cardiovascular Medicine, Tokyo Medical and Dental University (TMDU), Tokyo, 113-8510 Japan; 3 Laboratory of Molecular Neuroscience, Medical Research Institute, Tokyo Medical and Dental University (TMDU), Tokyo, 113-8510 Japan; 4 Laboratory of Gene Expression, Medical Research Institute, Tokyo Medical and Dental University (TMDU), Tokyo, 113-8510 Japan; 5 Department of Molecular Pathology, Graduate School of Medical and Dental Sciences, Tokyo Medical and Dental University (TMDU), Tokyo, 113-8510 Japan; 6 Graduate School of Frontier Biosciences, Osaka University, Suita, Osaka 565-0871 Japan

**Keywords:** Cardiomyopathies, Atrial fibrillation

## Abstract

Dilated cardiomyopathy (DCM) is a fatal heart disease characterized by left ventricular dilatation and cardiac dysfunction. Recent genetic studies on DCM have identified causative mutations in over 60 genes, including *RBM20*, which encodes a regulator of heart-specific splicing. DCM patients with *RBM20* mutations have been reported to present with more severe cardiac phenotypes, including impaired cardiac function, atrial fibrillation (AF), and ventricular arrhythmias leading to sudden cardiac death, compared to those with mutations in the other genes. An RSRSP stretch of RBM20, a hotspot of missense mutations found in patients with idiopathic DCM, functions as a crucial part of its nuclear localization signals. However, the relationship between mutations in the RSRSP stretch and cardiac phenotypes has never been assessed in an animal model. Here, we show that *Rbm20* mutant mice harboring a missense mutation S637A in the RSRSP stretch, mimicking that in a DCM patient, demonstrated severe cardiac dysfunction and spontaneous AF and ventricular arrhythmias mimicking the clinical state in patients. In contrast, *Rbm20* mutant mice with frame-shifting deletion demonstrated less severe phenotypes, although loss of RBM20-dependent alternative splicing was indistinguishable. RBM20^S637A^ protein cannot be localized to the nuclear speckles, but accumulated in cytoplasmic, perinuclear granule-like structures in cardiomyocytes, which might contribute to the more severe cardiac phenotypes.

## Introduction

Dilated cardiomyopathy (DCM) is a fatal cardiac disease characterized by enlargement of the cardiac chambers and impaired systolic function^1^. A recent study reported that the prevalence of DCM is estimated to be 1 in 250–500 people^2^. Among the cases of idiopathic DCM, 20–35% are familial, with autosomal dominant inheritance in most cases^3^, and over 60 genes have been identified as causative genes for DCM^4^.

*RBM20*, encoding RNA binding motif protein 20 (RBM20), was identified as one of the causative genes for DCM^5^. RBM20 is a major regulator of heart-specific alternative splicing of the *TTN* gene, which is found to be most frequently mutated in patients with idiopathic DCM (approximately 20–25%)^6,7^. The *TTN* gene has the largest number of exons (364 in humans) and titin, a sarcomeric protein encoded by the *TTN* gene, is the largest known protein in vertebrates^8^. In an *Rbm20* mutant rat strain lacking nearly all the *Rbm20* exons, the shortest cardiac titin isoform N2B is not expressed. A longer isoform N2BA is predominant in heterozygotes, and an aberrantly giant isoform N2BA-G is exclusively expressed in homozygotes^9^. This indicates that RBM20 is a key regulator of *Ttn* pre-mRNA processing in the heart. Since titin-based passive tension is negatively correlated with its molecular size^10–12^, the ratio of the titin isoforms is considered to impact the passive stiffness of the myocardium and the progression of DCM^13–15^. It is therefore assumed that *RBM20* mutations cause DCM phenotypes through altered splicing of the RBM20-regulated genes^9^.

Extensive searches for mutations in *RBM20* in patients with idiopathic DCM revealed a hotspot of missense mutations in a highly conserved RSRSP stretch, within an arginine/serine (RS)-rich region and not in the RNA-binding domains^5,9,16–20^. Recently, the RSRSP stretch has been found to be critical for the nuclear localization of RBM20, and mutations in this stretch resulted in the loss of RBM20-dependent alternative splicing^21^.

Clinically, the features of DCM with an *RBM20* mutation were reported as (1) the severely compromised cardiac systolic function leading to the need for heart transplantation at a very young age compared to that in DCM caused by mutations in other genes^22^, (2) higher incidence of sustained ventricular arrhythmias and sudden cardiac death than that in patients with a mutation in *TTN*^23^, and (3) higher incidence of atrial fibrillation (AF) than in patients with other idiopathic DCM^16^.

Cardiac functions of *Rbm20* have been analyzed in mutant mice in which only an RNA-recognition motif (RRM) domain is deleted^24^ or exons 4 and 5 are deleted for a frame-shift^23^. Homozygotes of these *Rbm20* mutations demonstrated severe splicing defects in *Ttn* and other target genes; however, the cardiac dysfunctions was mild^23,24^, as seen in the *Rbm20* mutant rat strain^9^. Despite the phenotypic discrepancies between patients of DCM with a heterozygous *RBM20* missense mutation and the homozygous *Rbm20* mutant animals, the consequences of the mutations in the RSRSP stretch have not yet been assessed in an animal model.

We compared the cardiac phenotypes between *Rbm20* knock-in and knock-out mice. We found that the *Rbm20* mutant mice harboring a missense mutation in the RSRSP stretch mimicking a mutation in a patient with DCM, showed severe cardiac dysfunction with atrial and ventricular arrhythmias, mimicking clinical patients. The mutant RBM20 protein is specifically localized to perinuclear granule-like structures in the cytoplasm of the cardiomyocytes, which might lead to the more severe cardiac phenotypes.

## Results

### ***Rbm20***^***S637A/S637A***^ and ***Rbm20***^***KO/KO***^ mice lose RBM20-dependent alternative splicing

**Figure 1:**
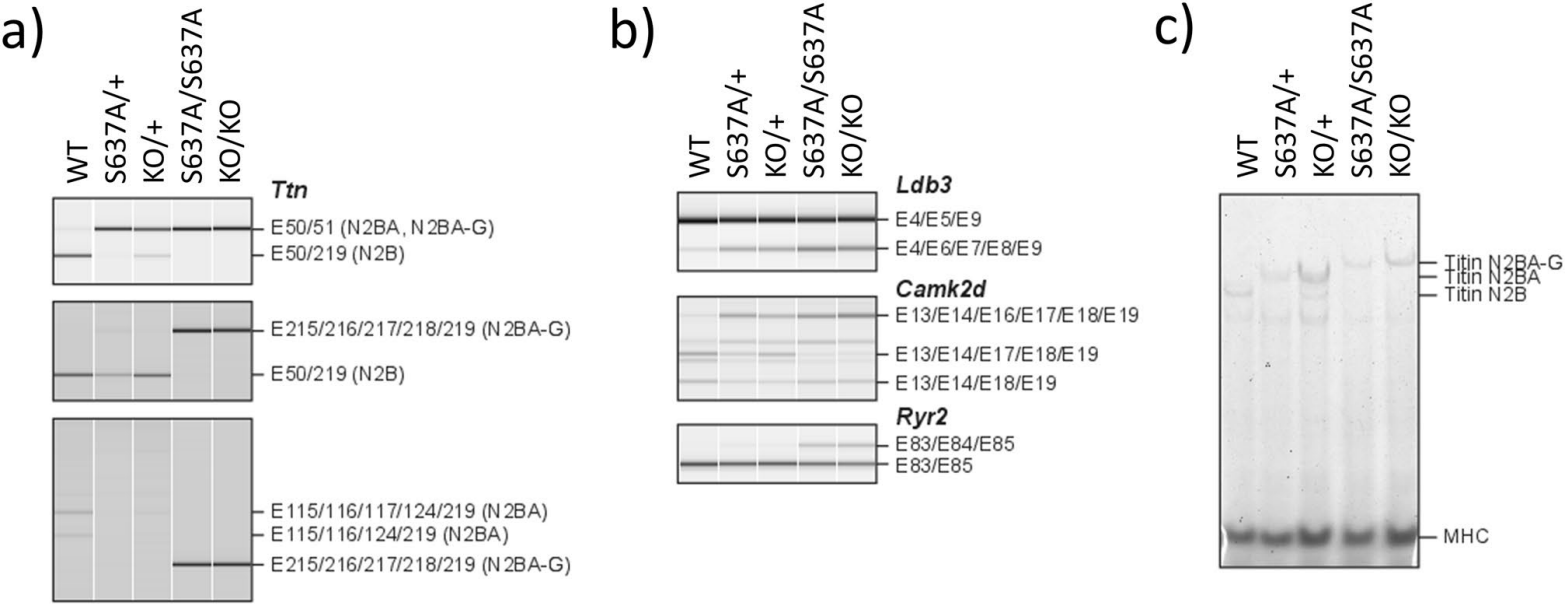
*Rbm20*^*S637A/S637A*^ and *Rbm20*^*KO/KO*^ mice are defective in RBM20-dependent alternative splicing. (**a**) RT-PCR analysis of *Ttn* mRNAs with exon (E) 50 forward, E51 reverse and E219 reverse primers (top), E50 forward, E215 forward and E219 reverse primers (middle) and E115 forward, E215 forward and E219 reverse primers (bottom). Splicing patterns of the PCR products and names of corresponding titin isoforms are indicated on the right. (**b**) RT-PCR analysis of *Ldb3* (top), *Camk2d* (middle), and *Ryr2* (bottom) mRNAs. Gel-like images by Bioanalyzer (Agilent) are shown (**a**,**b**). (**c**) Vertical SDS-agarose gel electrophoresis and CBB staining of cardiac proteins from the hearts of 4-week-old *Rbm20* mutant mice. Genotypes of the individual mice are indicated above. Titin isoforms (N2B, N2BA and N2BA-G) and myosin heavy chain (MHC) are indicated. n = 3 mice (**a**–**c**), each showing similar results. Full length original images are shown in Supplementary Information.

To understand the discrepancies in cardiac dysfunctions between patients with DCM and rodent models, we generated *Rbm20*^*S637A*^ knock-in^21^ and *Rbm20* knock-out mice by CRISPR/Cas9-mediated genome editing (Supplementary Information Fig. S1). We first analyzed the alternative splicing of known RBM20-regulated genes *Ttn*, *Ldb3*, *Camk2d*, and *Ryr2*^23,24^ in the ventricles, by reverse transcription (RT)-polymerase chain reaction (PCR). The splicing patterns of these genes in *Rbm20*^*S637A/S637A*^ and *Rbm20*^*KO/KO*^ mice were drastically different from those in the wild-type (WT), and were indistinguishable between the *Rbm20*^*S637A/S637A*^ and the *Rbm20*^*KO/KO*^ mice (Fig. 1a,b). Consistent with the results of RT-PCR, agarose gel electrophoresis of titin proteins revealed exclusive expression of the N2BA-G isoform of titin, which harbors all exons between exons 50 and 219 in both *Rbm20*^*S637A/S637A*^ and *Rbm20*^*KO/KO*^ mice (Fig. 1c). RT-PCR analysis of the ventricular RNAs from *Rbm20*^*S637A/*+^ and *Rbm20*^*KO/*+^ mice revealed intermediate effects on alternative splicing of the *Ttn, Ldb3* and *Camk2d* genes (Fig. 1a,b), and protein analysis confirmed the predominant expression of the N2BA isoform of titin (Fig. 1c). These results indicated that RBM20-dependent alternative splicing was completely dysregulated in both *Rbm20*^*S637A/S637A*^ and *Rbm20*^*KO/KO*^ mice, which is consistent with previous reports on *Rbm20* mutant animals^9,21,24^.

### *Rbm20*^*S637A/S637A*^ mice show more severe cardiac phenotypes

**Figure 2:**
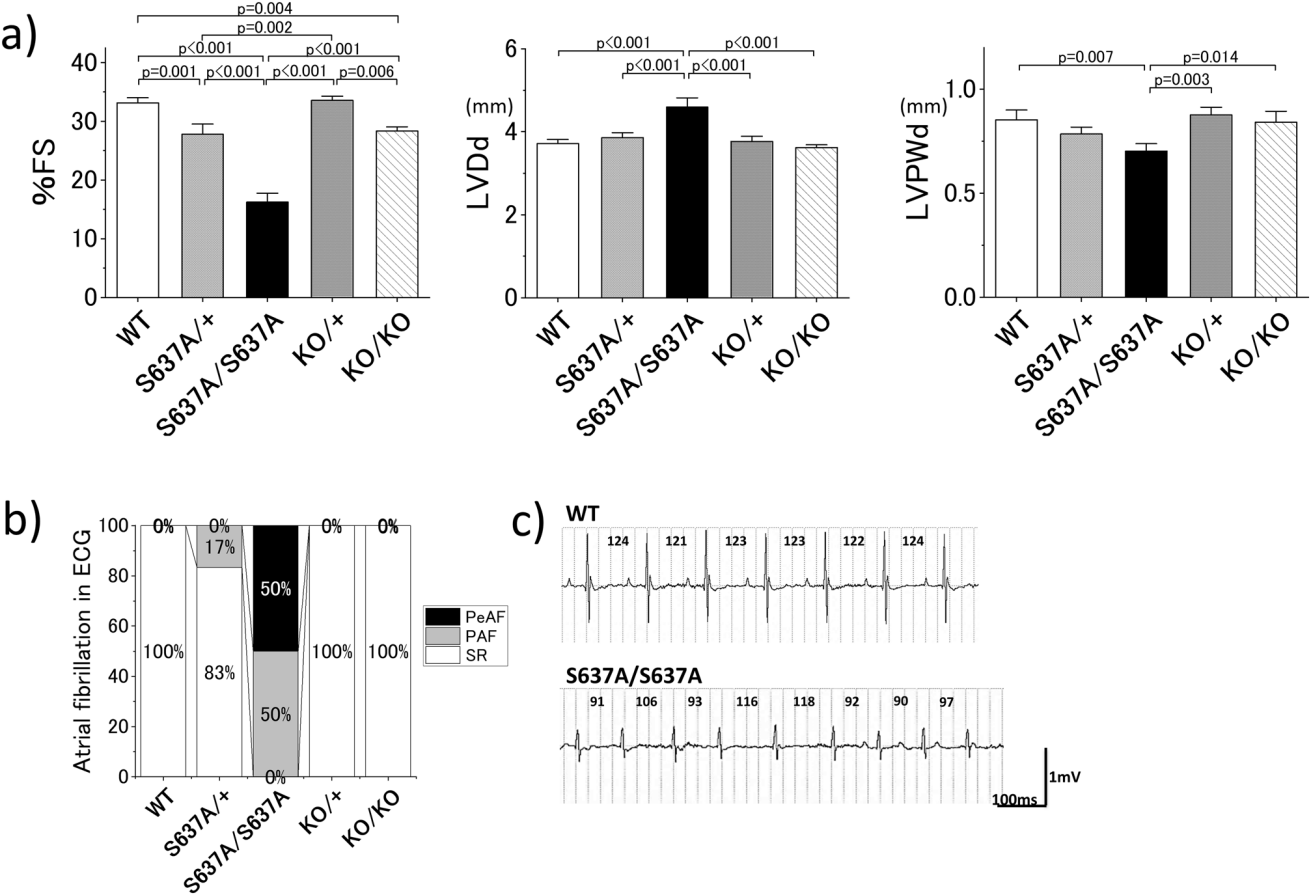
UCG and ECG at the age of 12–16 weeks reveal DCM-like phenotypes and AF in *Rbm20*^*S637A/S637A*^ mice. (**a**) Measured value of %FS, LVDd and LVPWd by UCG. n = 6 each. *p* values with statistical significance after Tukey's HSD test are indicated. Error bars, SEM. (**b**) Proportions of mice with AF in body-surface ECG. n = 6 each. PeAF, Persistent atrial fibrillation; PAF, paroxysmal atrial fibrillation; SR, sinus rhythm. (**c**) Representative ECG from WT and *Rbm20*^*S637A/S637A*^ mice. Values in the ECG indicate RR intervals. Note that the *Rbm20*^*S637A/S637A*^ mouse demonstrated AF with irregular RR intervals.

To assess the cardiac phenotypes of the *Rbm20* mutant mice, we performed ultrasound echocardiography (UCG) and electrocardiography (ECG) at the age of 12–16 weeks. In the UCG analysis, *Rbm20*^*KO/KO*^ mice showed slightly but significantly lower fractional shortening (%FS) compared to that in the WT mice (28.3 ± 1.2% vs 33.1 ± 1.4%, *p* = 0.002). *Rbm20*^*S637A/S637A*^ mice had far lower %FS (16.2 ± 2.5%, *p* < 0.001 compared to WT) than that in others (Fig. 2a and Supplementary Information Fig. S2). Left ventricular end-diastolic diameter (LVDd) was also significantly larger, and the end-diastolic left ventricular posterior wall thickness (LVPWd) was significantly thinner in the *Rbm20*^*S637A/S637A*^ mice (4.59 ± 0.36 mm and 0.70 ± 0.05 mm, respectively) compared to that in the *Rbm20*^*KO/KO*^ (3.62 ± 0.30 mm, *p* < 0.001, and 0.84 ± 0.08 mm, *p* < 0.001, respectively) and WT mice (3.71 ± 0.15 mm, *p* < 0.001, and 0.85 ± 0.07 mm, *p* < 0.001, respectively) (Fig. 2a). We also performed the same assessment on *Rbm20*^*S637A/*+^ and *Rbm20*^*KO/*+^ mice. Slightly but significantly lower %FS was found in the *Rbm20*^*S637A/*+^ mice compared to that in the WT, whereas the *Rbm20*^*KO/*+^ mice showed normal cardiac function comparable to the WT (Fig. 2a and Supplementary Information Fig S2). In the ECG analysis, all six *Rbm20*^*S637A/S637A*^ mice and one of the six *Rbm20*^*S637A/*+^ mice surprisingly demonstrated spontaneous AF, which was not seen in any of the *Rbm20*^*KO/KO*^*, Rbm20*^*KO/*+^*,* or WT mice (Fig. 2b, c). Our ECG and UCG assessments thus revealed more severe DCM-like phenotypes, including AF, in *Rbm20*^*S637A/S637A*^ mice than in the *Rbm20*^*KO/KO*^ mice, despite their indistinguishable defects in RBM20-dependent alternative splicing.

### Impairment of cardiac function is the primary pathological change in ***Rbm20***^***S637A/S637A***^ mice

**Figure 3:**
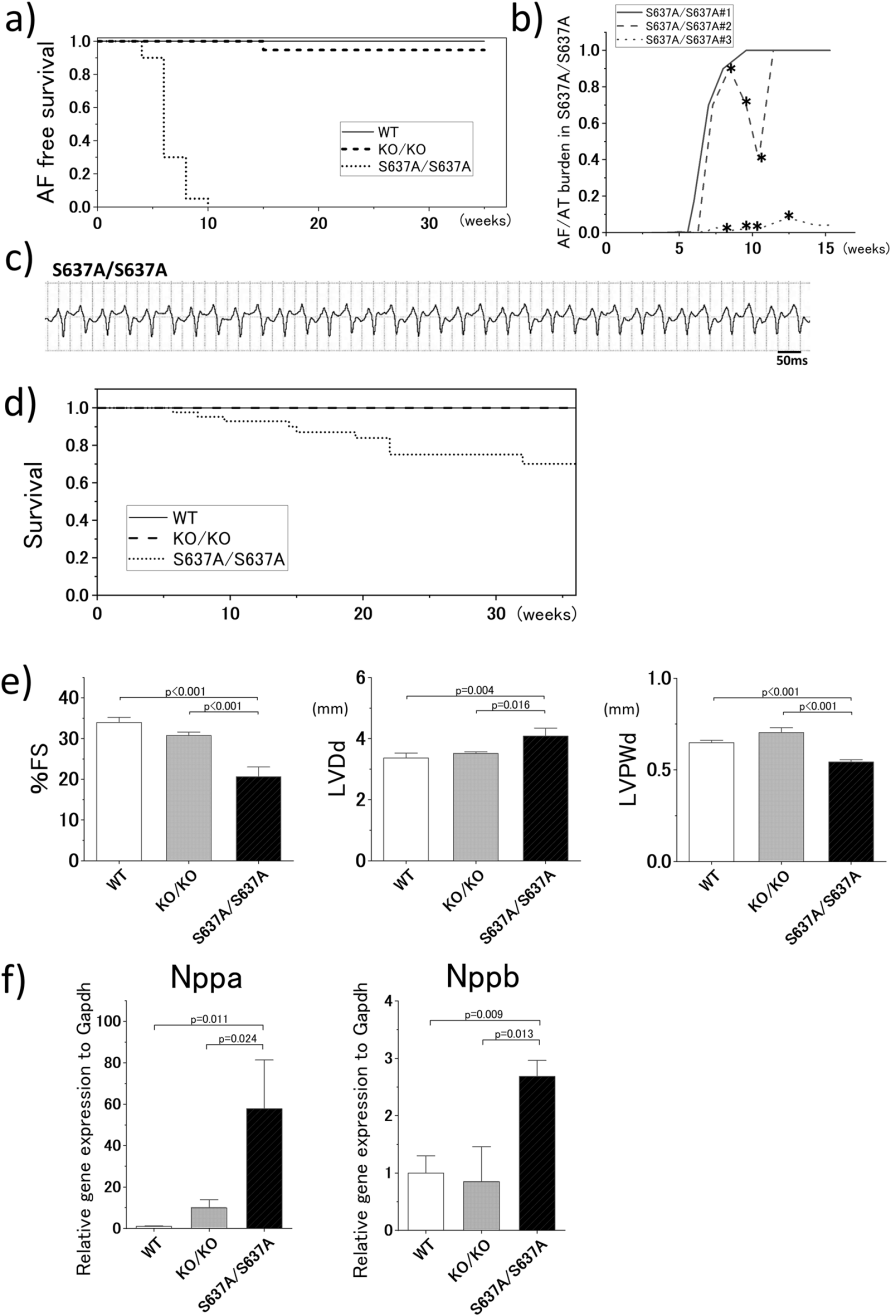
*Rbm20*^*S637A/S637A*^ mice show high prevalence of AF and VT, and severe cardiac dysfunction precedes the AF onset. (**a**) AF-free survival curve. n = 20–24 each. (**b**) Burden of atrial tachycardia (AT) and AF in *Rbm20*^*S637A/S637A*^ mice revealed by telemetry ECG. Three mice were analyzed. Asterisks indicate records in which VT was also observed in the telemetry ECG. (**c**) Spontaneous sustained VT with tachycardia cycle length of 50 ms observed in an *Rbm20*^*S637A/S637A*^ mouse with telemetry ECG. (**d**) Survival curve. n = 20–40 each. (**e**) Measured values of %FS, LVDd and LVPWd by UCG at the age of 4 weeks. n = 6 each. (**f**) Gene expression of *Nppa* and *Nppb* in the heart assessed by qPCR at the age of 4 weeks. n = 3 each. *p* values with statistical significance after Tukey's HSD test are indicated. Error bars, SEM.

Impaired cardiac function can cause and exacerbate AF and vice versa. To determine the primary change among the cardiac phenotypes, we checked the ECG of the *Rbm20* mutant mice every two weeks until the age of 12 weeks, and every four weeks thereafter. Body-surface ECG revealed that *Rbm20*^*S637A/S637A*^ mice started developing AF at the age of approximately 4 weeks and the presence of AF was evident in all the *Rbm20*^*S637A/S637A*^ mice at 10 weeks of age (Fig. 3a). Only one out of 20 (5%) *Rbm20*^*KO/KO*^ mice developed AF at 16 weeks of age, while the WT mice did not develop AF (Fig. 3a).

To precisely evaluate the onset of AF in *Rbm20*^*S637A/S637A*^ mice, we performed ambulatory ECG using telemetry ECG in three *Rbm20*^*S637A/S637A*^ mice. AF was first observed at the age of approximately 4 weeks (Fig. 3b and Supplementary Information Fig. S3), which is in line with the body-surface ECG findings. In two of the three mice, the burden of atrial arrhythmias significantly increased between 6–12 weeks of age, and AF persisted throughout the day by the age of 12 weeks. The third mouse still showed sinus rhythm (SR) and paroxysmal AF (PAF) at the age of 16 weeks. During the telemetry ECG, spontaneous, sustained, and self-terminating ventricular tachycardia (VT) and ventricular fibrillation (VF) were also observed in two of the three mice (Fig. 3b, c and Supplementary Information Fig. S4). During the VT episode, the mice suffered syncope, and VT sometimes occurred as an incessant form in these mice (Supplementary Information Fig. S4). In the survival curve analysis, sudden unexpected death was observed in approximately 30% of the *Rbm20*^*S637A/S637A*^ mice during the 36 weeks of follow-up (Fig. 3d).

Evaluation of the cardiac function by UCG revealed significantly lower %FS, larger LVDd, and lower LVPWd in *Rbm20*^*S637A/S637A*^ mice at the age of 4 weeks (Fig. 3e), when AF was not observed in the body-surface ECG. These results indicate that cardiac dysfunction preceded the onset of AF as the primary cardiac change. Gene expression levels of *Nppa* and *Nppb*, which are the biomarkers for heart failure, were assessed by quantitative PCR (qPCR), and both were significantly higher in the *Rbm20*^*S637A/S637A*^ mice than in the WT and *Rbm20*^*KO/KO*^ mice at 4 weeks of age (Fig. 3f), supporting the onset of functional phenotypes.

### Morphological and histological changes in the heart of ***Rbm20***^***S637A/S637A***^ mice are consistent with DCM

**Figure 4:**
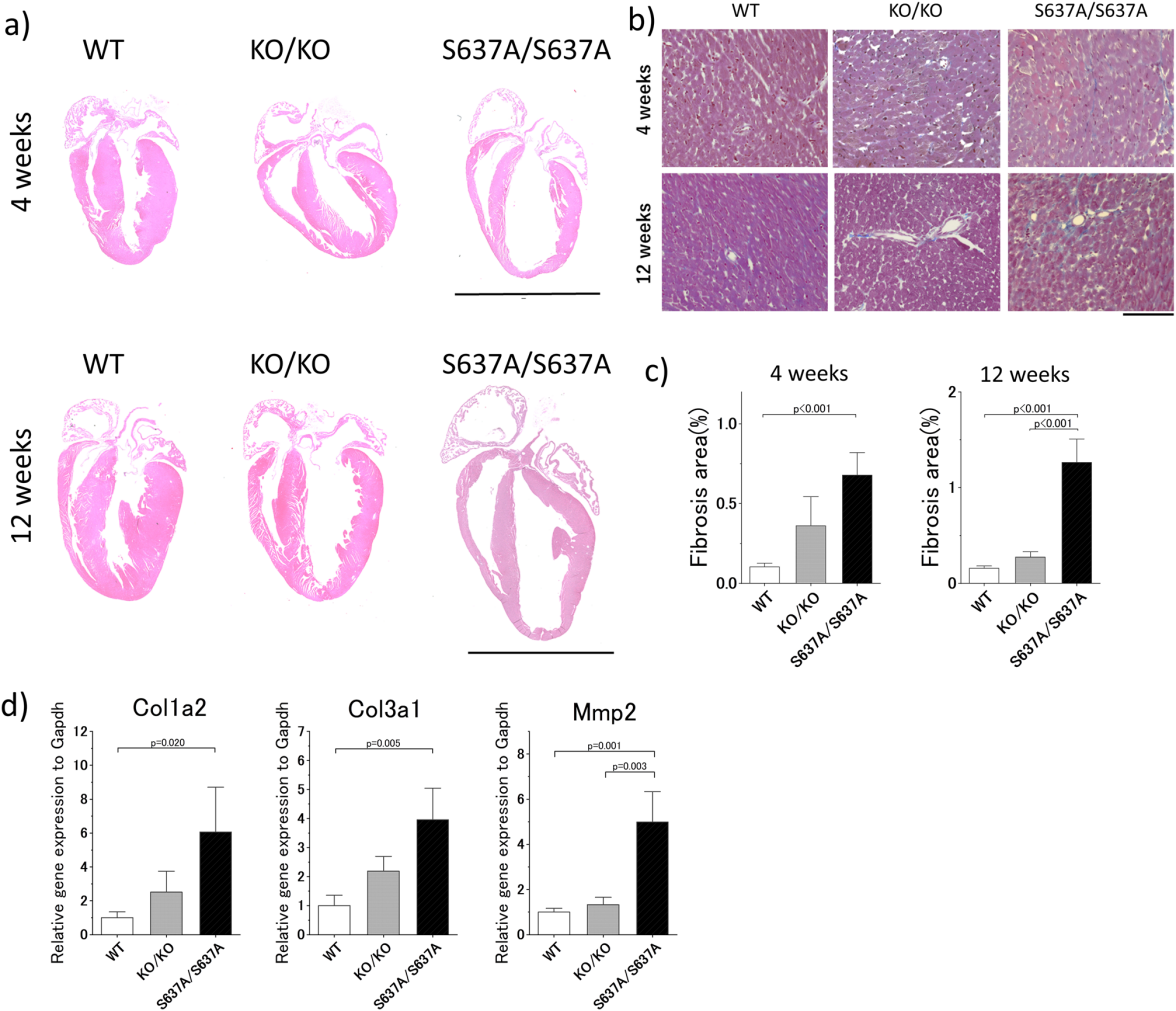
Histological assessment of the hearts from *Rbm20*^*S637A/S637A*^ mice shows morphological changes compatible with DCM. (**a**) Representative images of H&E staining at the age of 4 weeks (top) and 12 weeks (bottom). Scale bars, 5 mm. (**b**) Representative images of Masson’s trichrome staining at the age of 4 weeks (top) and 12 weeks (bottom). Scale bar, 100 µm. (**c**) Proportions of fibrosis area measured with the images of the Masson’s trichrome staining. n = 4–6 each. *p* values with statistical significance after Tukey's HSD test are indicated. Error bars, SEM. (**d**) qPCR analysis of genes related to cardiac fibrosis at the age of 4 weeks. n = 3 each. *p* values with statistical significance after Tukey's HSD test are indicated. Error bars, SEM.

Considering that *RBM20*^*S635A*^, a missense mutation in humans corresponding to *Rbm20*^*S637A*^ in mice, is causative for DCM^9^, morphological and histological changes in the heart were evaluated in the *Rbm20*^*S637A/S637A*^ mice. Hematoxylin and eosin (H&E) staining revealed that the hearts of the *Rbm20*^*S637A/S637A*^ mice showed enlarged cardiac chambers and thinning of the ventricular wall at the age of 4 weeks (Fig. 4a), which was compatible with DCM cardiac phenotypes. Cardiac fibrosis was also found in *Rbm20*^*S637A/S637A*^ mice with Masson’s trichrome staining (Fig. 4b, c). qPCR analysis revealed upregulation of the genes related to cardiac fibrosis, including *Col1a2, Col3a1,* and *Mmp2* (Fig. 4d), which was consistent with the histological observation. At 12 weeks of age, these morphological and fibrotic LV changes were exacerbated in the *Rbm20*^*S637A/S637A*^ mice (Fig. 4a–c). These typical DCM changes were barely observed in the hearts of *Rbm20*^*KO/KO*^ mice (Fig. 4a–c).

### ECG in *Rbm20*^*S637A/S637A*^ mice shows abnormal electrophysiological properties in both the atrium and ventricle

**Figure 5:**
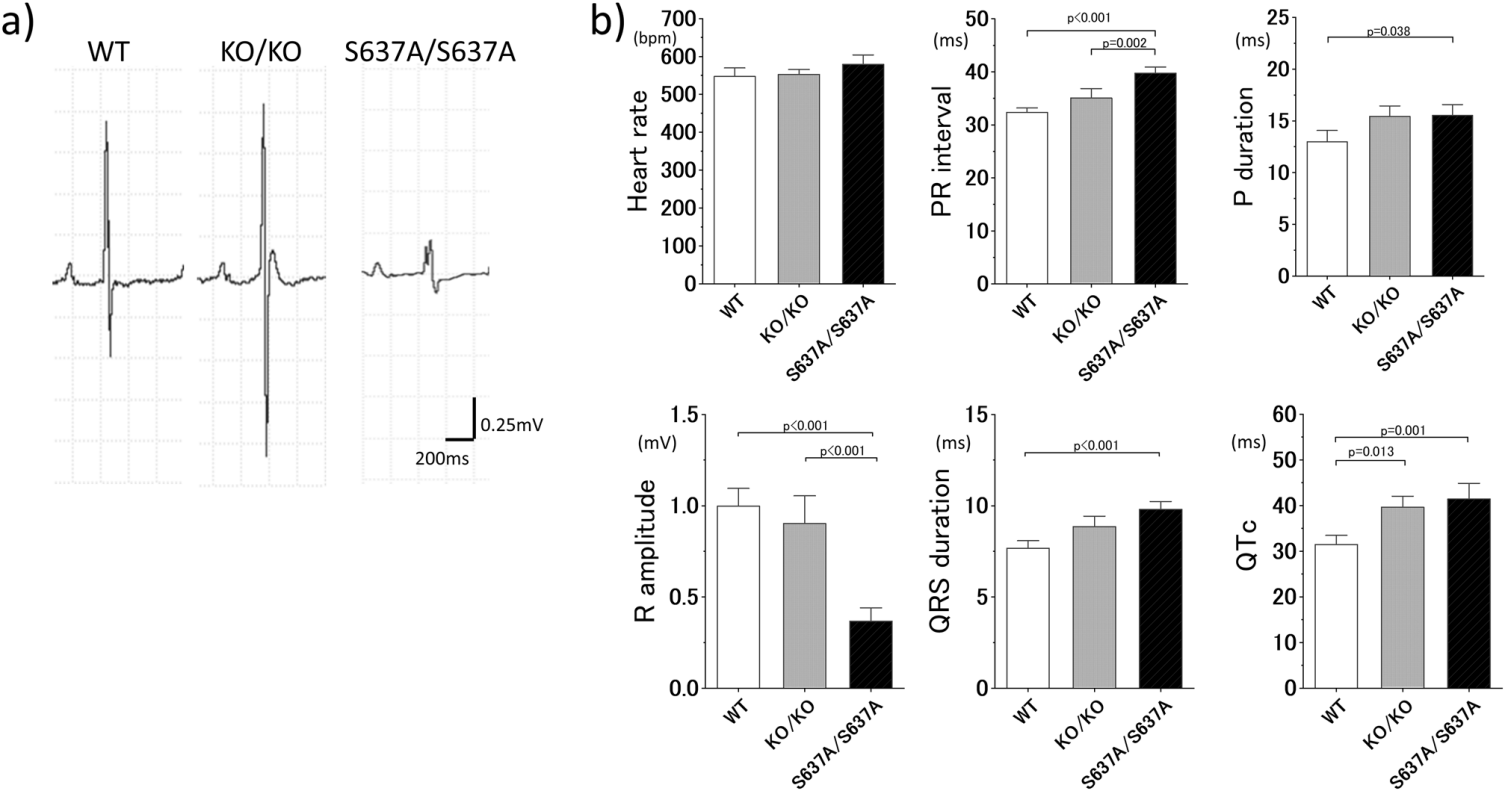
Body-surface ECG at the age of 4 weeks reveals atrial and ventricular electrophysiological abnormalities in *Rbm20*^*S637A/S637A*^ mice. (**a**) Representative images of the ECG. Genotypes are indicated at the top. (**b**) Measured ECG parameters. n = 7–8 each. *p* values with statistical significance after Tukey's HSD test are indicated. Error bars, SEM.

Body-surface ECG at the age of 4 weeks revealed significantly prolonged P wave duration, PR interval, QRS duration and heart rate-corrected QT interval (QTc), as well as significantly smaller R amplitude in the *Rbm20*^*S637A/S637A*^ mice compared to those in the WT mice as shown in Fig. 5 (15.5 ± 2.2 vs 13.0 ± 2.3 ms, *p* = 0.038 for P wave duration, 39.8 ± 2.5 vs 32.3 ± 1.8 ms, *p* < 0.001 for PR interval, 9.8 ± 0.9 vs 7.7 ± 0.9 ms, *p* < 0.001 for QRS duration, 41.4 ± 7.2 vs 31.5 ± 4.2 ms, *p* = 0.001 for QTc). As shown in Supplementary Information Table S1, the coefficient of variation for these ECG parameters was acceptable and consistent for each measurement, indicating the reproducibility of the ECG results. These observations are compatible with those observed in patients with DCM except for the QTc interval^25^. In particular, the prolongation of the P wave duration and PR interval reflects conduction disturbance in the atria, which indicates that the substrate of AF was present before the development of AF.

*Rbm20*^*KO/KO*^ mice showed the same tendency in the ECG parameters as the *Rbm20*^*S637A/S637A*^ mice, yet the differences were not significant except for the QTc interval (Fig. 5). These observations in the *Rbm20*^*KO/KO*^ mice were consistent with those in a previous report^23^.

### RNA-seq reveals upregulation of the skeletal muscle genes and downregulation of the DCM-related genes in the heart of ***Rbm20***^***S637A/S637A***^ mice

**Figure 6:**
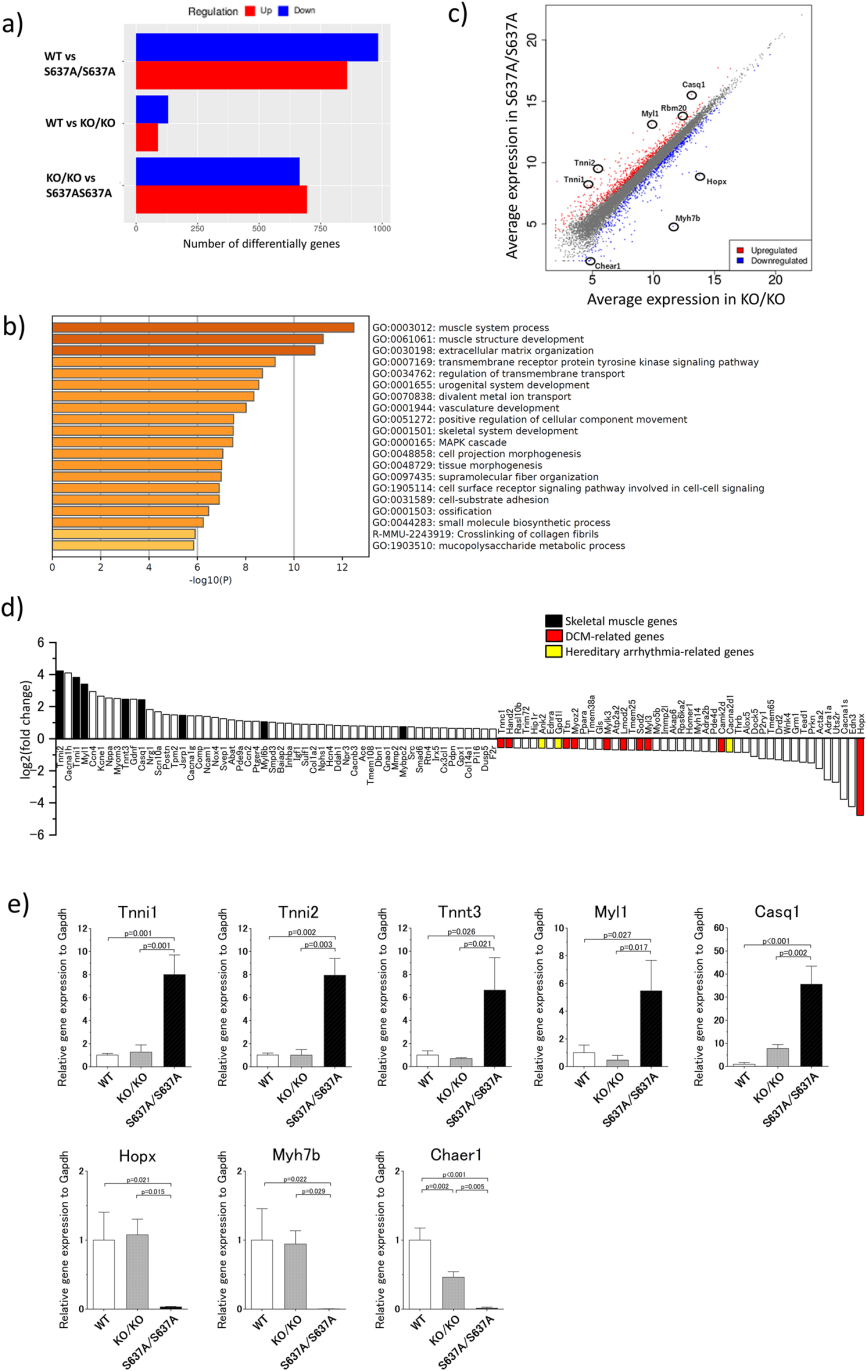
Gene expression profile of *Rbm20*^*S637A/S637A*^ mice is distinct from those of WT and *Rbm20*^*KO/KO*^ mice. Poly(A)^+^ RNAs extracted from ventricles of WT and *Rbm20* mutant mice (n = 3 each) were subjected to RNA-seq analysis. (**a**) Numbers of DEGs between indicated genotypes of mice. A cut-off of a FDR < 0.05 and gene expression fold change > 1.5 or < 0.667 were applied. (**b**) Enrichment analysis of *Rbm20*^*S637A/S637A*^ vs *Rbm20*^*KO/KO*^. Bar colors represent p values. (**c**) A scatter plot for *Rbm20*^*S637A/S637A*^ vs *Rbm20*^*KO/KO*^. Each dot represents each gene. Skeletal muscle genes and DCM-related genes top-ranked among the DEGs are indicated. (**d**) Relative expression levels of DEGs between *Rbm20*^*S637A/S637A*^ and *Rbm20*^*KO/KO*^ mice with a GO term ‘muscle system process’ (GO:0003012). Skeletal muscle genes, DCM-related genes, and causative genes of hereditary arrhythmia are colored as indicated. (**e**) qPCR analysis of DEGs top-ranked in the RNA-seq analysis. n = 3 each. *p* values with statistical significance after Tukey's HSD test are indicated. Error bars, SEM.

To compare the gene expression profiles in *Rbm20*^*S637A/S637A*^ and *Rbm20*^*KO/KO*^ mice, we performed massive RNA sequencing (RNA-seq) of poly(A)^+^ RNAs from the heart, at 4 weeks of age. We detected 1840 differentially expressed genes (DEGs) between the WT and *Rbm20*^*S637A/S637A*^ mice (857 upregulated and 983 downregulated genes), whereas only 219 genes were differentially expressed between the WT and *Rbm20*^*KO/KO*^ mice (89 upregulated and 130 downregulated genes) (Fig. 6a and Supplementary Information Fig. S5a,b). A total of 1359 transcripts were differentially expressed between the *Rbm20*^*S637A/S637A*^ and *Rbm20*^*KO/KO*^ mice (694 upregulated and 664 downregulated in the *Rbm20*^*S637A/S637A*^ mice). Gene ontology analysis revealed differences in the biological processes especially in the muscle system process, muscle structure development and extracellular matrix organization between the *Rbm20*^*S637A/S637A*^ and *Rbm20*^*KO/KO*^ mice (Fig. 6b), whereas there was a difference with low *p* values in genes related to muscle contraction between the WT and *Rbm20*^*KO/KO*^ mice (Supplementary Information Fig. S5c, d). We also found that skeletal muscle genes including *Tnni1*, *Tnni2*, *Tnnt3*, *Myl1*, *Casq1*, *Jsrp1*, *Myl6b,* and *Mybpc2* were significantly upregulated, and DCM-related genes including *Hopx*, *Camk2d*, *Myl3*, *Sod2*, *Lmod2*, *Mylk3*, *Myoz2*, *Ttn*, *Hand2*, *Tnnc1*, *Myh7b,* and *Ctf1* were significantly downregulated in the heart of the *Rbm20*^*S637A/S637A*^ mice (Fig. 6c–d, Supplementary Information spreadsheet 1, 2). For some of the DEGs, we tested the mRNA and protein expression levels (Fig. 6e, Supplementary Information Fig. S6), and found them to be consistent with the results of RNA-seq. Additionally, a long non-coding RNA related to cardiac hypertrophy, *Chaer1,* and genes related to hereditary arrhythmic disorders, including *Ank2*, *Gpd1l*, and *Cacna2d1,* were downregulated in the *Rbm20*^*S637A/S637A*^ mice (Fig. 6c–e). These results confirmed that the gene expression profile of the *Rbm20*^*S637A/S637A*^ mice is distinct from that of the *Rbm20*^*KO/KO*^ mice.

### RBM20^**S637A**^ protein accumulates in cytoplasmic, perinuclear granule-like structures

**Figure 7:**
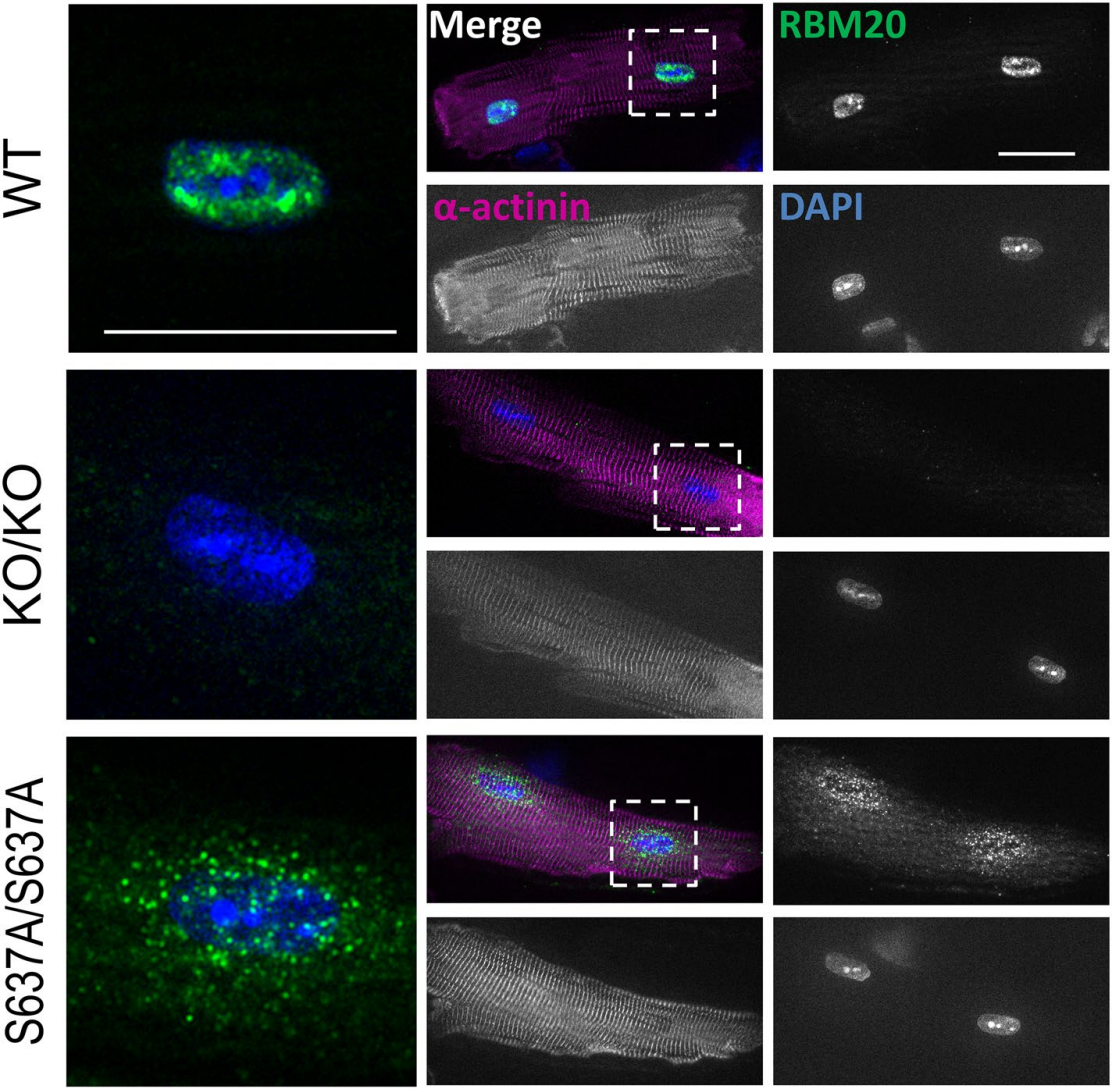
RBM20^S637A^ protein accumulates in cytoplasmic, perinuclear granule-like structures in cardiomyocytes. Isolated cardiomyocytes from WT, *Rbm20*^*KO/KO*^ and *Rbm20*^*S637A/S637A*^ mice were stained with anti-RBM20 and anti-α-actinin antibodies and DAPI. Representative images are shown with pseudo colors. Dotted regions around nuclei are magnified and shown on the left as merged images. Scale bars, 25 µm. n = 3 mice, each showing similar results.

Remarkable DCM-like phenotypes of *Rbm20*^*S637A/S637A*^ mice described above were not observed in the *Rbm20*^*KO/KO*^ mice or other *Rbm20* mutant animals reported so far, indicating that the phenotypes are caused by expression of the mutant RBM20 protein and not just by loss-of-function of the *Rbm20* gene. We therefore investigated the subcellular localization of RBM20^S637A^ protein in the cardiomyocytes of *Rbm20*^*S637A/S637A*^ mice. In a previous study, we reported the cytoplasmic localization of FLAG-tagged RBM20^S637A^ protein in heterologous cells^21^. In this study, we created a custom-made antibody against the C-terminus of mouse RBM20 protein, and detected the endogenous RBM20 proteins by immunofluorescence staining of the isolated cardiomyocytes (Fig. 7). Cytoplasmic, perinuclear granule-like RBM20 signals were detected in the *Rbm20*^*S637A/S637A*^ cardiomyocytes (Fig. 7), whereas the RBM20 signals were localized only to the nuclear speckles in the WT cardiomyocytes (Fig. 7), as has been reported previously^26,27^. No specific signals were detected in the *Rbm20*^*KO/KO*^ cardiomyocytes (Fig. 7), confirming the specific detection of endogenous RBM20 proteins on staining. In cardiomyocytes from *Rbm20*^*S637A/*+^ mice, RBM20 signals were present in both nuclear speckles and cytoplasmic perinuclear granule-like structures (Fig. S7). These results indicated that RBM20^S637A^ protein cannot be localized to the nuclear speckles but is specifically accumulated in the cytoplasmic, perinuclear granule-like structures in cardiomyocytes.

## Discussion

In this study, we demonstrated for the first time that.

1. *Rbm20*^*S637A/S637A*^ mice and not *Rbm20*^*KO/KO*^ mice showed severe cardiac dysfunction with a high prevalence of AF and VT mimicking the clinical DCM phenotypes with a mutation in *RBM20*.
2. The hearts of the *Rbm20*^*S637A/S637A*^ mice showed upregulation of the skeletal muscle genes and downregulation of the cardiomyopathy-related genes.
3. RBM20^S637A^ protein accumulates in the cytoplasmic, perinuclear granule-like structures in the *Rbm20*^*S637A/S637A*^ cardiomyocytes.

One notable characteristic of the *Rbm20*^*S637A/S637A*^ mice is the development of AF (Fig. 2b, c). It is generally very difficult to induce AF in mice, and spontaneous AF is very unusual, especially at 4 weeks of age. In previous studies, few transgenic strains have been reported to develop spontaneous AF^28,29^. In contrast to previous AF mouse models, *Rbm20*^*S637A/S637A*^ is the first mouse strain to show spontaneous, persistent AF with only a single nucleotide substitution, mimicking a clinical patient. Moreover, a recent genome-wide association study revealed *RBM20* as one of the AF-related genes in the general population^30^, although the exact *RBM20* variant associated with AF is yet to be determined. Therefore, the use of *Rbm20*^*S637A/S637A*^ mice is likely to be a great advantage in future studies on the mechanism of AF development in vivo.

A previous study using cardiomyocytes from *Rbm20 null* mice revealed increased spontaneous release of Ca^2+^ from the sarcoplasmic reticulum due to increased L-type Ca^2+^ current, intracellular Ca^2+^ overload, and increased sarcoplasmic reticulum Ca^2+^ content, likely caused by splicing disturbance of *Camk2d*^23^, although the *Rbm20 null* mice did not show VT in vivo^23^. Therefore, we speculated that this abnormal Ca^2+^ handling could contribute to the development of VT in the *Rbm20*^*S637A/S637A*^ mice. Moreover, it is also well known that Ca^2+^ leaks from the sarcoplasmic reticulum, and intracellular Ca^2+^ overload in the atrial cardiomyocytes is crucial for the development of AF^31,32^. Therefore, similar to that in the ventricular cardiomyocytes, the abnormal Ca^2+^ handling by loss of RBM20-dependent splicing might contribute to atrial arrhythmogenicity as observed in both *Rbm20*^*S637A/S637A*^ and *Rbm20*^*KO/KO*^ mice (Fig. 3a). However, this mechanism cannot completely explain the higher incidence of ventricular arrhythmias in the *Rbm20*^*S637A/S637A*^ mice compared to that in the *Rbm20*^*KO/KO*^ mice.

Prolongation of the QTc interval in *Rbm20 null* mice was previously reported^23^, and was considered to be due to the loss of RBM20-dependent alternative splicing. In this study, the *Rbm20*^*S637A/S637A*^ mice showed QTc prolongation similar to that in the *Rbm20*^*KO/KO*^ mice, and we speculated that the prolongation of QTc interval in the *Rbm20*^*S637A/S637A*^ mice might be likewise affected by splicing defects. The study also mentioned that the difference in potassium currents between humans and mice might cause a discrepancy in the QTc prolongation between RBM20 mutation carriers and the *Rbm20 null* mice^23^.

Previous in vivo studies on the function of RBM20 were conducted on *Rbm20 null* mice^23^ or rats^9^, or mice with a deletion of the RRM domain^24^. These *Rbm20* mutant animals or the *Rbm20*^*KO/KO*^ mice in this study did not show severe cardiac dysfunction or development of AF. Considering the indistinguishable splicing defects (Fig. 1) and the identical genetic background of the *Rbm20*^*S637A/S637A*^ and *Rbm20*^*KO/KO*^ mice, the phenotypic differences between them indicate that the *Rbm20*^*S637A*^ mutation is not only a loss-of-function mutation in terms of the splicing defects, but also a gain-of-function mutation that leads to the severe DCM phenotypes. In particular, both a homozygous and heterozygous *Rbm20*^*S637A*^ mutation caused impaired cardiac function and AF in adulthood (Fig. 2). These observations are consistent with the fact that all reported *RBM20* missense mutations in DCM patients were heterozygous^20^.

A molecular feature of the *Rbm20*^*S637A*^ mutation is the presence of RBM20^S637A^ protein, which accumulates in the perinuclear granule-like structures of unknown characters in the cardiomyocytes. Nuclear clearance and cytoplasmic deposition of a nuclear RNA-binding protein RBM20 in non-dividing cells in the DCM model are analogous to a pathological hallmark of neurodegenerative diseases in which predominantly nuclear RNA-binding proteins such as TAR DNA-binding protein 43 kDa (TDP-43) form cytoplasmic inclusions. TDP-43 proteinopathy is an established disease concept, and is well known as the cause of amyotrophic lateral sclerosis (ALS), frontotemporal lobar degeneration (FTLD), and other neurodegenerative diseases^33^. Mutations in the *TARDBP* gene encoding TDP-43 as well as in other genes lead to the cytoplasmic aggregation of TDP-43 in the neurons. A small subset of familial ALS is associated with missense mutations in the *MATR3* gene, a paralogue of *RBM20*^34,35^. Considering that the *Rbm20*^*S637A*^ mutation specifically led to the DCM phenotypes as demonstrated in this study, and the analogy to neurodegenerative diseases, it is reasonable to assume that cytoplasmic RBM20^S637A^ protein contributes to the development of DCM. However, the pathogenesis of the TDP-43 proteinopathy remains elusive, despite extensive studies^36–38^, partly because TDP-43 is essential for viability. Future studies on the pathogenesis of RBM20^S637A^ would lead to a better understanding of the diseases caused by aggregation and/or altered functions of the RNA-binding proteins.

Comprehensive mRNA analysis using the hearts from *Rbm20*^*S637A/S637A*^ mice revealed misexpression of skeletal muscle genes and downregulation of DCM-causative genes compared to those from the WT and *Rbm20*^*KO/KO*^ mice (Fig. 6 and Supplementary Information Fig. S5). Misexpression of skeletal muscle genes has been previously reported to cause cardiac malfunction^39–41^. *Hopx*, *Myl3*, *Sod2*, *Lmod2*, *Mylk3*, *Myoz2*, *Ttn*, *Hand2*, *Tnnc1*, *Myh7b* and *Ctf1* have been reported as definitive or putative DCM genes^4,42–52^, and a long noncoding RNA gene *Chaer1* was also reported to be an important factor in cardiac hypertrophy^53^. Therefore, downregulation of these genes is consistent with the severe DCM-like phenotypes of the *Rbm20*^*S637A/S637A*^ mice, although it is currently unclear whether this is the cause or the result of the phenotypes. The DEGs in the *Rbm20*^*S637A/S637A*^ mice included many other genes related to cardiac dysfunction such as *Slc5a1*^54^ and *Mylk4*^55^ (Supplementary Information spreadsheet 1, 2). With respect to arrhythmia, expressions of *Ank2*, a causative gene of long QT syndrome^56^, and *Gpd1l* and *Cacna2d1*, the causative genes of Brugada syndrome^57,58^, were affected, which might contribute to the arrhythmogenicity in the heart of the *Rbm20*^*S637A/S637A*^ mice.

There are some limitations in this study. First, we did not address the precise mechanism by which RBM20^S637A^ protein causes DCM-like phenotypes, including AF and VT. Although this study clearly demonstrated that altered splicing of RBM20-regulated genes was not alone responsible for causing the phenotypes, it is still unclear whether altered splicing is involved in the pathogenesis. In particular, in terms of arrhythmogenicity, it is unclear whether there is a common mechanism for atrial and ventricular arrhythmias related to the RBM20^S637A^ protein. Further studies are necessary to understand the roles of the RBM20^S637A^ protein accumulation on the cytoplasmic structures and alternative splicing changes in the target genes in cardiac phenotypes. Second, although the development of AF is a unique feature in the *Rbm20*^*S637A/S637A*^ mice, the roles of the RBM20^S637A^ protein and impaired cardiac function in the development of AF are unclear. In addition to the *Rbm20*^*S637A/S637A*^ mice, one of the *Rbm20*^*KO/KO*^ mice developed AF spontaneously during long-term follow up (Fig. 3a), indicating that the loss of RBM20 also contributes to the development of AF. In addition, heart failure is a well-known factor for exacerbation of AF^59^. Thus, high AF prevalence in the *Rbm20*^*S637A/S637A*^ mice might be the consequence of heart failure, loss of RBM20-dependent alternative splicing, presence of cytoplasmic RBM20^S637A^ protein, and other unknown defects. To elucidate the primary effect of the *Rbm20*^*S637A*^ mutation on the development of AF, a mouse with atrium-specific RBM20^S637A^ expression will be required. Furthermore, the most common form of AF is not associated with cardiac dysfunction, and its molecular phenotype is different from that of AF with heart failure^60^. Therefore, another appropriate animal model is required for common AF. Third, the subcellular localization of RBM20 proteins has not been investigated in patients with clinical DCM, with and without a mutation in *RBM20*. To evaluate the relevance of the granule-like structures in *Rbm20*^*S637A/S637A*^ mice with the DCM phenotypes of the patients, further experiments using human biopsy samples or cardiomyocytes differentiated from patient-derived induced pluripotent stem cells (iPSCs) are necessary.

In conclusion, *Rbm20*^*S637A*^, a missense mutation in the RSRSP stretch of RBM20, leads to severe DCM-like phenotypes as well as AF and VT. *Rbm20*^*S637A/S637A*^ mice offer a novel path for future therapeutics as a unique mouse model for DCM and AF.

## Methods

### Animals

All animal experiments were in accordance with the guidelines for the Care and Use of Laboratory Animals published by National Research Council (The National Academy Presses, eighth edition, 2011), pre-approved and performed under the regulation of the Institutional Animal Care and Use Committee of Tokyo Medical and Dental University (Approval #A2019-105C5 and #A2019-178C2). Generation of the *Rbm20*^*S637A*^ knock-in allele was previously reported^21^. Briefly, the *Rbm20*^*S637A*^ allele was generated by utilizing a cloning-free CRISPR/Cas system^61^. The *Rbm20*^*KO*^ allele was obtained from the identical heterozygous founder mouse of the *Rbm20*^*S637A*^ allele as a result of non-homologous end joining that yielded a 26-bp deletion (Supplementary Information Fig. S1 ). Wild-type (WT) littermate mice were used as controls. All experiments were performed with male mice.

### Anti-RBM20 antibody generation

The polyclonal antibody against mouse RBM20 was generated in rabbit by using a synthetic C-terminal peptide PERGGIGPHLERKKL (amino acids 1185–1199, accession number NP_001164318) as antigen by Cosmobio. The anti-RBM20 antibody was sequentially purified with affinity columns coupled with protein A and the synthetic C-terminal peptide.

### Electrophoresis and staining of cardiac proteins

Vertical SDS-agarose gel electrophoresis of cardiac proteins from mice were performed essentially as described previously^21,62^. Protein samples were extracted from ventricular tissues with urea buffer (8 M urea, 2 M thiourea, 3% SDS w/v, 75 mM DTT and 0.03% bromophenol blue in 50 mM Tris–Cl, pH 6.8) and separated by using 1% Sea Kem Gold agarose (FMC bioproducts) gel. The proteins were detected by staining with CBB (Bio Craft). The images of the stained gels were captured with a scanner GT-X700 (Epson) and processed by using Photoshop CC (Adobe).

### Western blot

Protein samples were extracted from murine heart ventricular tissues with 10 mM Tris-Maleate buffer (pH 7.0). Protein concentration was measured by BCA Protein Assay Reagent Kit (Thermo Fisher Scientific). Twenty μg of protein samples were loaded on a 4–15% SDS-PAGE gel (Bio-Rad) for separation and transferred to PVDF membranes with Trans-Blot Turbo system (Bio-Rad). After blocking with Blocking One (Nacalai tesque), the membranes were incubated overnight at 4 °C with the custom-made anti-RBM20 affinity-purified antibody described above (1 µg/ml), anti-Tnni1 (1:500, rabbit polyclonal, Proteintech), anti-Tnni2 (1:500, rabbit polyclonal, Proteintech), or anti-GAPDH (1:10,000, mouse monoclonal, Santa Cruz Biotechnology). HRP-labeled secondary antibodies (1:10,000, Dako) were used for detecting the specific bands. Chemiluminescense signals (GE Healthcare) were detected by using iBright CL1500 (Thermo Fisher Scientific), and analyzed using iBright Analysis Software (Thermo Fisher Scientific).

### Electrocardiography and ultrasound echocardiography

Body-surface electrocardiography (ECG) and ultrasound echocardiography (UCG) were performed in a blinded manner as described previously^63^. ECG parameters were obtained by averaging those from three different ECGs. Coefficient of variation was calculated from them. QT interval was defined as an interval between the onset of the QRS complex and the end of the negative component of the T wave. QTc was calculated by the following formula: QTc = QT interval (ms)/√(RR interval (s) × 10). To assess the incidence of AF, 10-min ECG was performed at the age of 4, 6, 8, 10 and 12 weeks, and every 4 weeks after the age of 12 weeks. In this study, we defined AF as an irregular RR rhythm persisting longer than 5 s and not accompanying a clear P wave on ECG, paroxysmal AF (PAF) as self-terminating AF within 10 min, and persistent AF (PeAF) as AF persisting throughout 10-min ECG. Atrial tachycardia (AT) was defined as regular RR tachycardia distinguishable on ECG from sinus rhythm (SR).

### Telemetry ECG

A telemetry ECG transmitter (ETA-F10, DSI) was subcutaneously implanted into the back of the mouse^64^. A telemetry receiver was placed under the cage and the output signal of ECG was digitized with PowerLab (ADInstruments). Ambulatory ECG recording for 24 h was conducted every 1–2 weeks after the age of 3 weeks.

### RNA extraction and RT-PCR

Total RNAs were extracted from heart ventricular tissues by using RNeasy Mini Kit (QIAGEN) according to the manufacturer's protocol. cDNAs were synthesized by using High Capacity cDNA Reverse Transcription Kit (Thermo Fisher Scientific) or PrimeScript II 1st strand cDNA Synthesis Kit (Takara) from 1 μg of total RNAs. qPCR was performed with PowerSYBR Green Master Mix (Thermo Fisher Scientific) and custom-made primers. Expression levels were normalized with *Gapdh*. Semi-quantitative RT-PCR for analyzing splicing patterns was performed by using PrimeStarGXL (Takara) and primers previously described^21^. The PCR products were analyzed by utilizing Bioanalyzer 2100 Expert with DNA1000 Kit (Agilent). PCR primers used in this study were synthesized by Fasmac or Eurofins. Sequences of the primers are available in Supplementary Information Table S2.

### Histological assessment

Excised hearts were immersed in 10% buffered formalin and paraffin sections were subjected to H&E staining or Masson’s trichrome staining. Stained sections were photographed (DZ-710, Keyence), and fibrotic area was quantified by using ImageJ software (NIH). Proportion of fibrotic area was calculated as ratio of the fibrotic area to the total cross-sectional area.

### Cardiomyocyte isolation and immunofluorescence staining

Ventricular myocytes were isolated by using a published protocol^65^ with minor modification. Animals were pretreated with heparin (1000 units/kg body weight) and anesthetized with intraperitoneal infusion of 100 mg/kg of pentobarbital sodium. Hearts were excised, and cannulated aorta was fixed to a Langendorff apparatus. The hearts were perfused with Ca^2+^-free Tyrode’s solution (120 mM NaCl, 5.4 mM KCl, 1.2 mM MgSO_4_, 1.2 mM NaH_2_PO_4_ and 5.6 mM glucose in 20 mM NaHCO_3_, pH 7.4) for 5 min at 2 ml/min, followed by perfusion with Tyrode’s solution supplemented with 0.4 mg/ml Collagenase type II (GIBCO), 0.06 mg/ml Protease type XIV (Sigma-Aldrich), and 1 mg/ml fatty acid free BSA (Sigma-Aldrich) for 18 min. The perfusion solution was bubbled with 95% O_2_/5% CO_2_ and maintained at 37 °C. The ventricles were cut into several pieces and subjected to gentle agitation through a cell strainer (70 µm, Falcon) to separate the cardiomyocytes.

Isolated cardiomyocytes were fixed with 2% paraformaldehyde in PBS for 10 min. The cells were permeabilized with PBS containing 0.1% Triton X-100 for 30 min. After blocking, the cells were incubated with 1 µg/ml anti-RBM20 antibody (described above) and anti-α-actinin antibody (1:200, mouse monoclonal, Sigma-Aldrich) for 1 h at 37 °C, followed by incubation with Alexa488-conjugated goat anti-rabbit IgG (1:200, Invitrogen), Alexa647-conjugated goat anti-mouse IgG (1:200, Invitrogen) and DAPI for 30 min. Fluorescence images were captured by utilizing optimal sectioning with a digital microscope (BZ-X710, Keyence) equipped with a 100 × oil-immersion objective (NA = 1.45, Nikon).

### Statistical analysis

All data except RNA-seq are shown in mean ± standard error of the mean (SEM). Tukey's HSD test was used to compare more than 2 groups. Statistical analyses were performed with JMP10 (SAS Institute Inc.). A *p* value < 0.05 was considered as statistically significant.

### RNA-seq

After RNA extraction, NanoDrop (Thermo Fisher Scientific), and Bioanalyzer (Agilent) were used to check the purity, concentration, and RIN values. After poly(A)^+^ RNA selection with NEBNext Poly(A) mRNA Magnetic Isolation Module (New England Biolabs), sequencing libraries were constructed by using NEBNext Ultra Directional RNA Library Prep Kit for Illumina (New England Biolabs). The libraries were sequenced on Illumina Novaseq 6000 by Novogene. Paired-end, 150-nt reads were generated. The sequence data files have been submitted to DRA (Accession number: DRA010298).

### Analysis of RNA-seq data

The quality of obtained sequence data was evaluated using FastQC program (https://www.bioinformatics.babraham.ac.uk/projects/fastqc/) and trimmed by using the Trim Galore tool (https://www.bioinformatics.babraham.ac.uk/projects/trim_galore/). Then, the reads were mapped to the mouse reference genome (mm10) utilizing HISAT2^66^. Sequence alignment/map (.SAM) format files were converted into binary alignment/map (.BAM) format using SAMtools^67^. StringTie was used for transcript assembling^68^. Finally, the processed data was analyzed with integrated differential expression and pathway analysis (iDEP.91) (https://bioinformatics.sdstate.edu/idep/)^69^. In iDEP package, read count data were normalized by counts per million function in edgeR with a pseudo-count of 4. Normalized count data were transformed using the logarithm function, and transformed data was used for gene expression analysis based on DESeq2 package. A cut-off of a false discovery rate (FDR) < 0.05 and gene expression fold change > 1.5 or < 0.667 were applied. Metascape (https://metascape.org/) was used for the gene set enrichment analysis. A gene list for Metascape analysis was generated by iDEP.

## Supplementary information

Supplementary Information 1.

Supplementary Information 2.

Supplementary Information 3.

## Data Availability

The datasets generated during and/or analyzed during the current study are available from the corresponding authors on reasonable request.
