# Supplementary material for: A missense mutation in the RSRSP stretch of Rbm20 causes dilated cardiomyopathy and atrial fibrillation in mice
Source: Sci Rep. 2020 Oct 27;10:17894. doi: 10.1038/s41598-020-74800-8 (PMC7591520; doi:10.1038/s41598-020-74800-8)

Supplementary information for

A missense mutation in the RSRSP stretch of *Rbm20* causes dilated cardiomyopathy and atrial fibrillation in mice.

Kensuke Ihara*, Tetsuo Sasano, Yuichi Hiraoka, Marina Togo-Ohno, Yurie Soejima, Motoji Sawabe, Megumi Tsuchiya, Hidesato Ogawa, Tetsushi Furukawa, Hidehito Kuroyanagi*

*To whom correspondence should be addressed.

Kensuke Ihara, M.D. Ph.D. Tel: +81-3-5803-4950; E-mail: iharcvm@tmd.ac.jp, or

Hidehito Kuroyanagi, Ph.D. Tel: +81-3-5803-4695; E-mail: kuroyana.end@tmd.ac.jp

Table S1

Coefficient of variation (%) for ECG parameters

	Heart rate	PR interval	P duration	QRS interval	QTc	R amplitude
WT	2.6	3.9	7.0	3.8	5.3	3.1
Rbm20$^{KO/KO}$	2.5	3.1	6.8	2.3	5.1	2.8
Rbm20$^{S637A/S637A}$	2.1	4.4	7.3	3.0	5.8	6.2

Table S2

Sequences of primers used in this study

Gene	Primer sequence F	Primer sequence R
Nppa qPCR	gaagatgccggtagaagatgaggt	actctgggctccaatcctgtcaat
Nppb qPCR	gtccagcagagacctcaaaa	aggcagagtcagaaactgga
Tnni1 qPCR	tccacaacaccagagagatcaagg	gcatggcatcggctgagacacg
Tnni2 qPCR	tgcagagttcctgcacttca	ccgccgagaatctgagaagg
Tnnt3 qPCR	attgaccaagcccagaagca	gaaggctggagagtgcagag
Myl1 qPCR	gcaacaggaggacttcaagg	tctcttcattgctggggttc
Casq1 qPCR	cttcgctaccttcgacagca	gggttgatctcctgtgctcc
Chaer1 qPCR	tccaatgagggaagcgaagc	gtccgatgccagttccagtt
Myh7b qPCR	gcctctgcggacattgatag	gggcagctggaagatcact
Hopx qPCR	ggaggagcagacgcagaaatg	tggctccctagtccgtaacag
Gapdh qPCR	tgtgatgggtgtgaaccacgagaa	gagcccttccacaatgccaaagtt
Col1a2 qPCR	ccccgggactcctggactt	gctccgacacgccctctctc
Col3a1 qPCR	ggaaaggatggagagtcaggaa	cattgcgtccatcaaagcct
Mmp2 qPCR	agaaggacaagtggtccgcgtaaa	ctgcattgccacccatggtaaaca
Ryr2 splicing	gttgtcacgatgaagaagatgatg	ctttgctggcactgatagtctg
Camk2d splicing	aagggcgccatcttgacaac	tcaaagtccccattgttgat
Ldb3 splicing	tccaagcggcctattcccatc	tgtattctgtcccggtcatctg
Rbm20 splicing	acatgctccgggaagctgacag	ggatagtgtttccggtcatgtg
Ttn splicing	gagatccaaggagccccccaa (E50F)	tctgccagatgatctcaatcac (E51R)
	cacagagccatatgaagaacccta (E115F)	gcaccacctcttcctttggc (E219R)
	aaagcaaccattaccattggtaga (E215F)	

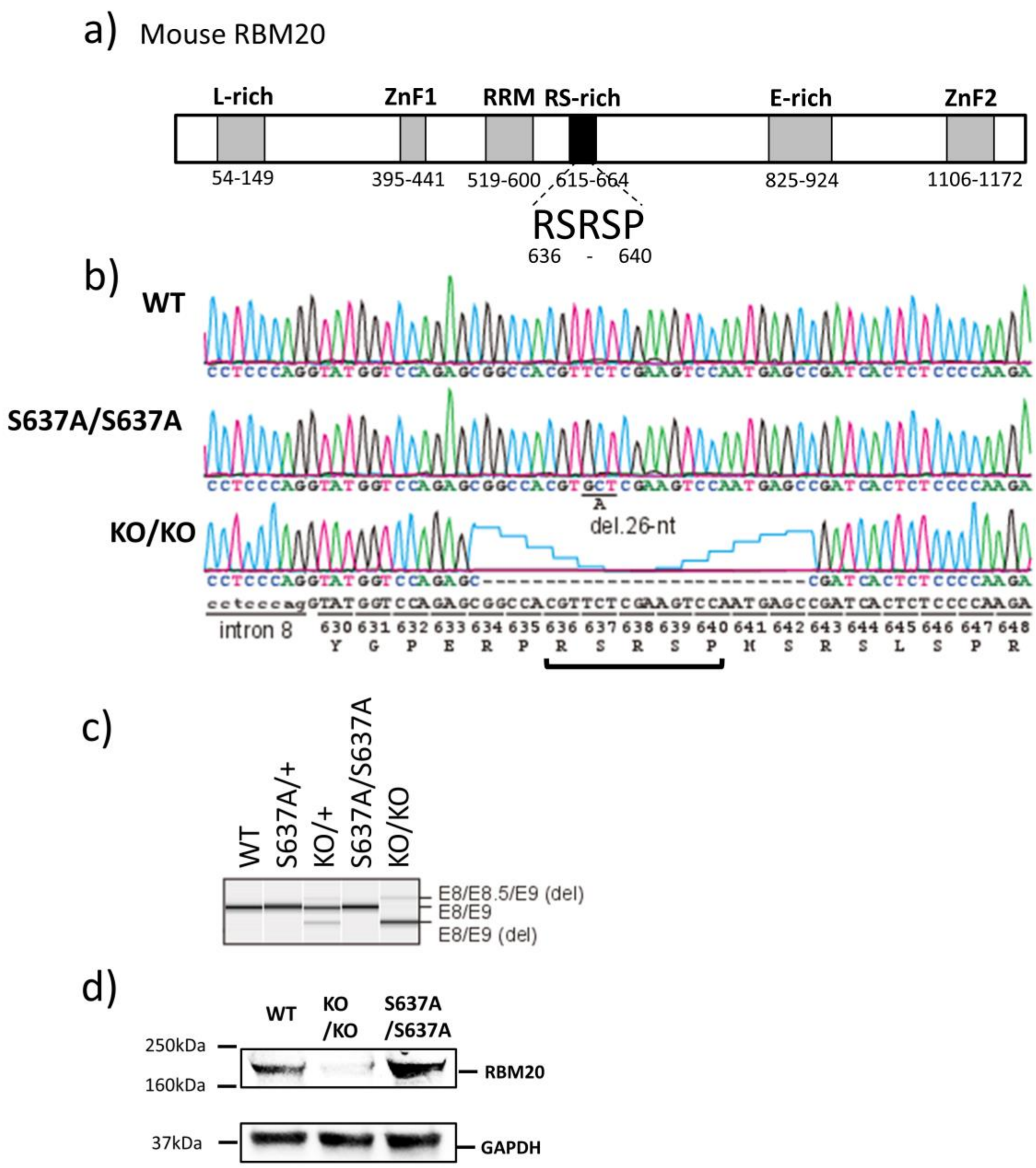

Figure S1. Generation of *Rbm20* mutant mice.

a) Schematic structure of the mouse RBM20 protein. Names and positions of the domains are indicated. E-rich, glutamate-rich region; L-rich, leucine-rich region; RRM, RNA-recognition motif domain; RS-rich, arginine/serine-rich region; ZnF, zinc finger domains. The RSRSP stretch is positioned at aa 636–640 in the RS-rich region.

b) Genomic sequence analysis of *Rbm20* intron 8 and exon 9 from WT, *Rbm20*$^{S637A/S637A}$, and *Rbm20*$^{KO/KO}$ mice. The RSRSP stretch is indicated.

c) RT-PCR analysis of exons 8-9 of *Rbm20* mRNAs. Splicing patterns of the PCR products are indicated on the right. Deletion of a 26-nt stretch in exon 9 leads to a frameshift and nonsense-mediated mRNA decay (NMD) of mRNAs from the *Rbm20*^{KO} allele. Note that a 41-nt pseudo exon (E8.5) located between exon 8 and exon 9 is occasionally included in mRNAs from the *Rbm20*^{KO} allele. Inclusion of E8.5 restores the reading frame, and the mRNA with E8.5 escapes NMD. However, it unlikely produces functional RBM20 protein because it is minor and lacks the RSRSP stretch.

d) Western blot of RBM20 using the heart tissue.

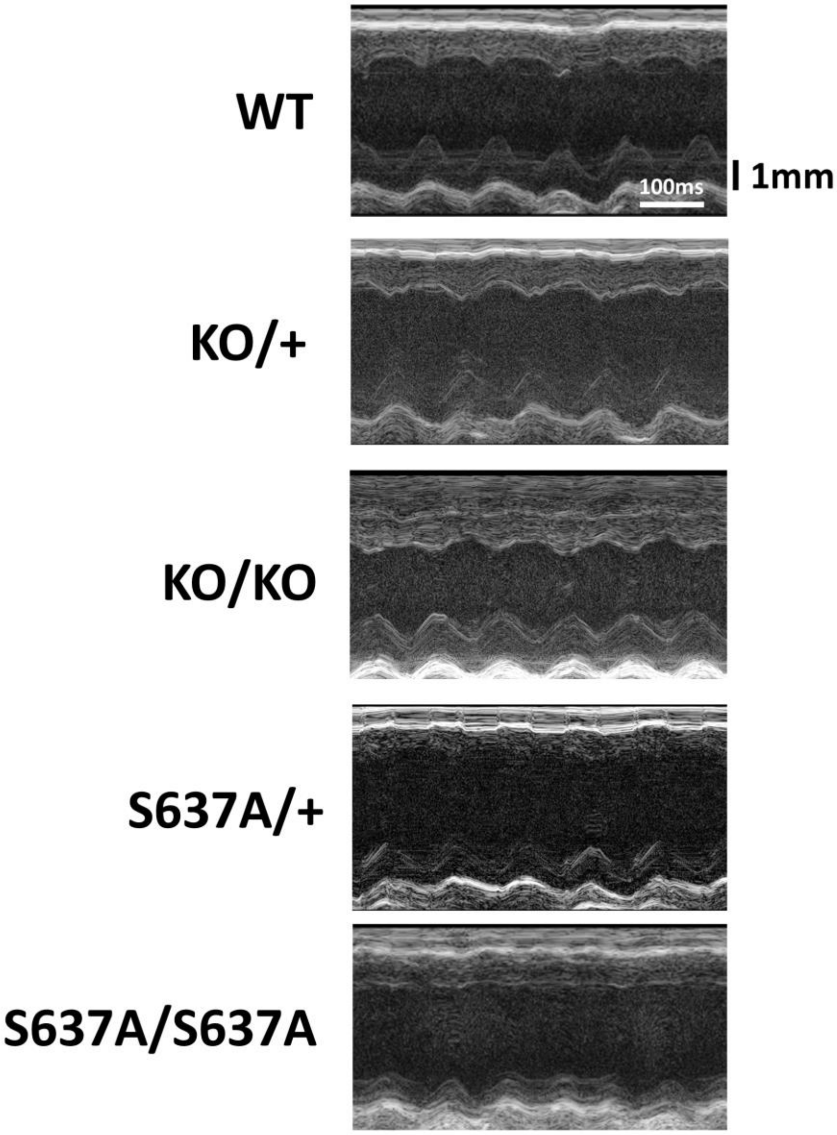

Figure S2. Representative UCG images at the age of 12-16 weeks.

Genotypes of the mice are indicated on the left.

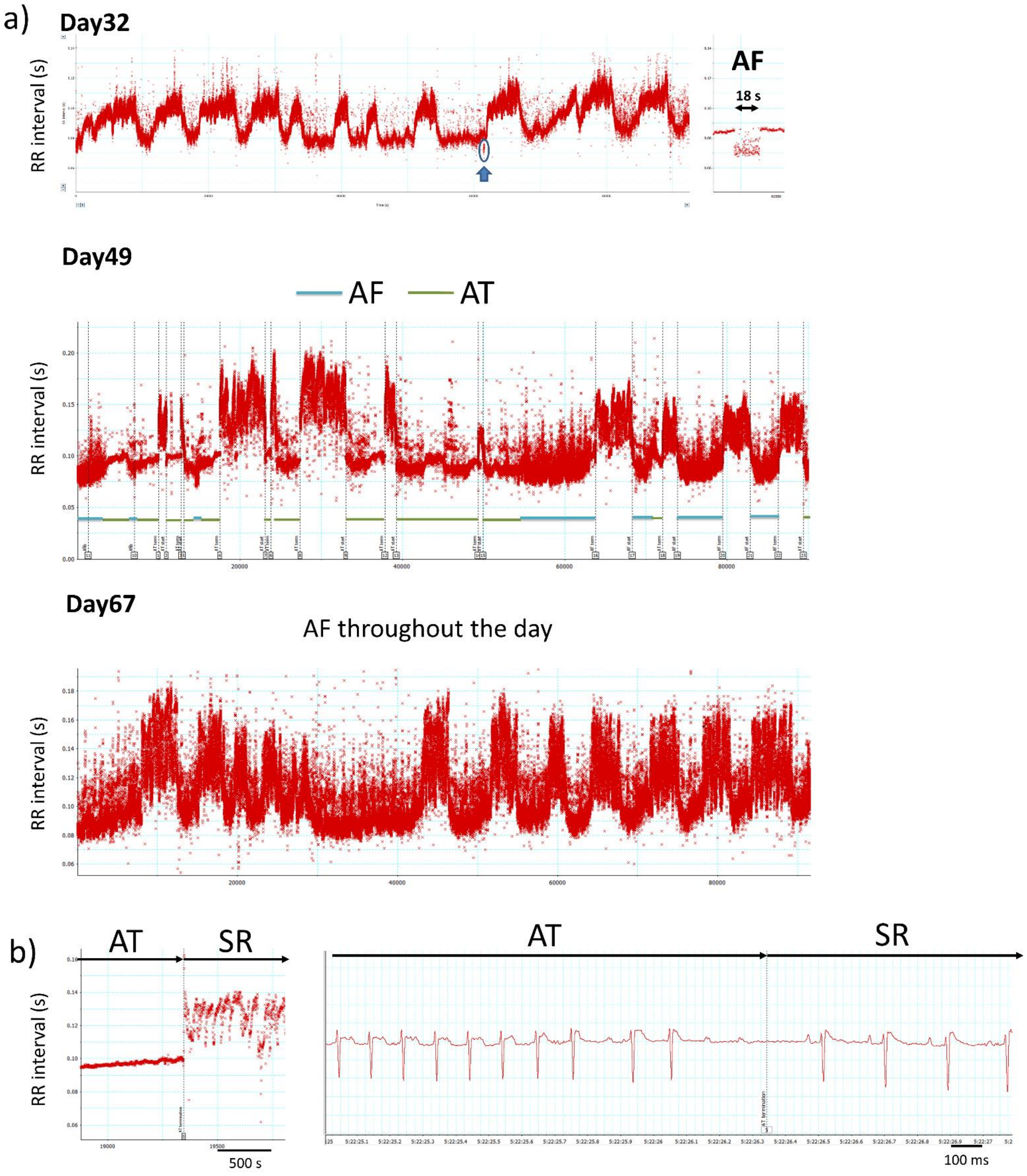

Figure S3. Representative images of RR plot from 24-hour telemetry ECG of an *Rbm20*$^{S637A/S637A}$ mouse.

a) A short AF episode (blue arrow, irregular RR tachycardia) of 18 s was documented for the first time at day 32 in this mouse (top panel). The RR plot during AF episode is enlarged in the right panel. At day 49 (middle panel), both AT and AF were observed as indicated. At day 67 (bottom panel), SR was no longer be observed, and AF persisted.

b) A representative RR plot and enlarged ECG of AT and its termination are shown. AT was confirmed by stable RR interval which was distinguishable from sinus rhythm as shown here. P wave morphology is also useful for determining AT.

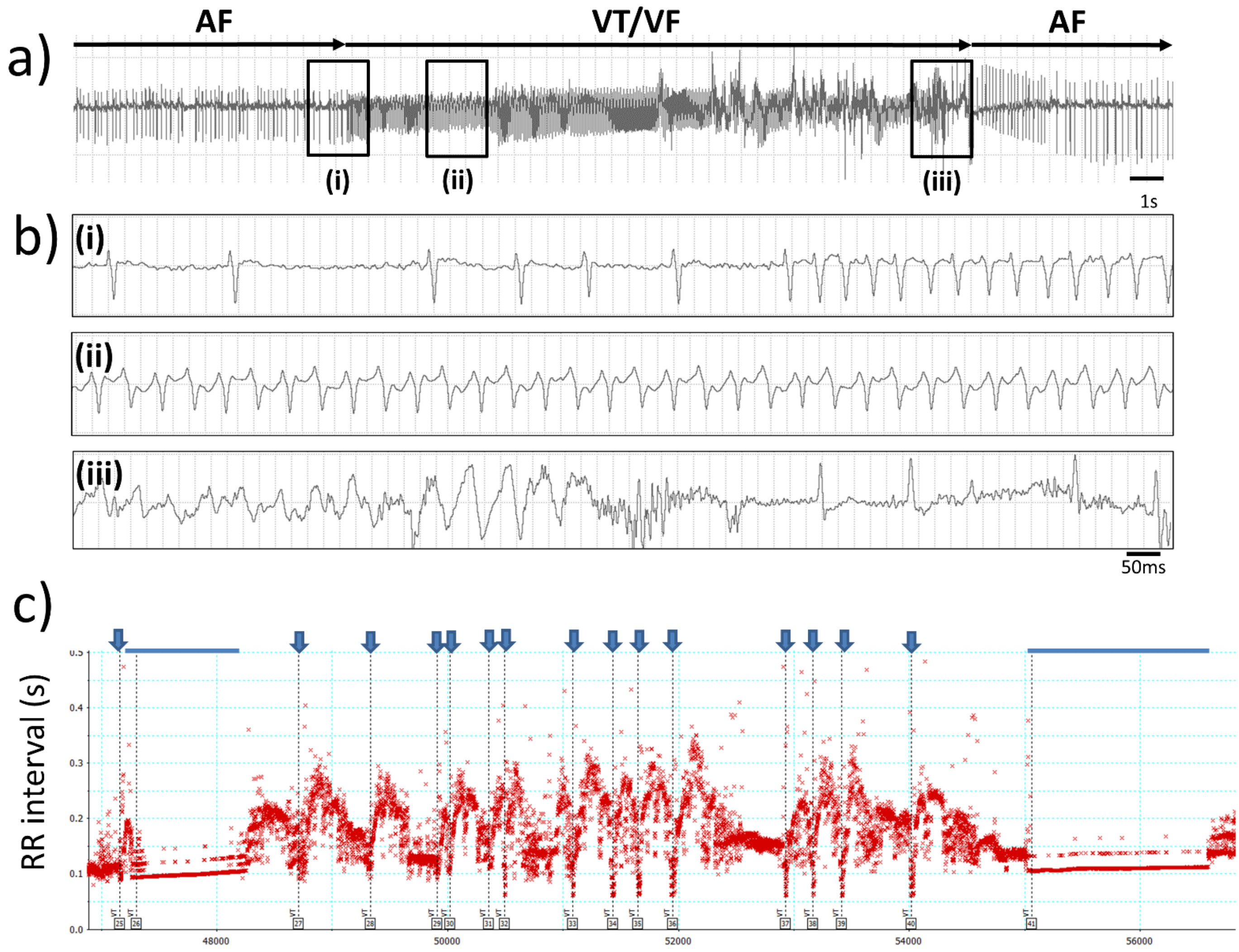

Figure S4. Representative telemetry ECG during the VT and VF in an *Rbm20*$^{S637A/S637A}$ mouse.

a) A compressed ECG of a whole episode of one VT/VF for approximately 20 s is shown. Baseline rhythm was AF. VT was converted to VF and finally self-terminated.

b) Enlarged ECGs at the onset, during and at the termination of VT/VF are shown. Indicated numbers correspond to those in panel a). (ii) is the same image as in Fig. 3c.

c) An RR plot of the incessant VT episodes on day 59 is shown. A total of 17 VT episodes including those lasting for 16 min and 36 min were seen within 200 min. Blue arrows and bars indicate VT events.

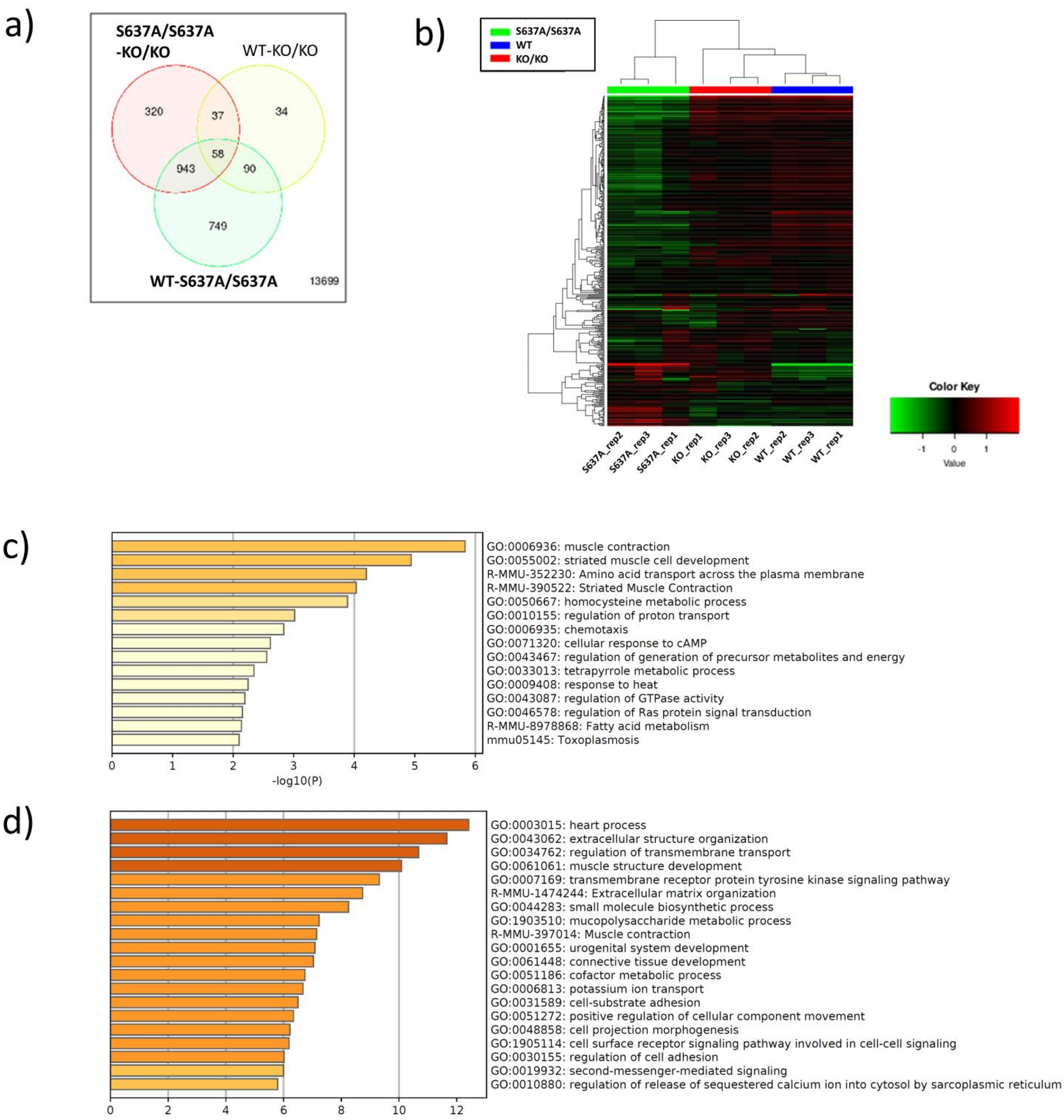

Figure S5. Results of RNA-seq using Poly(A)+ RNAs extracted from ventricles.

a) A Venn diagram showing numbers of DEGs between indicated genotypes of mice.

b) A heat map of the top 400 variable genes in the RNA-seq analysis.

c, d) Enrichment analysis of DEGs between WT vs *Rbm20*$^{KO/KO}$ (c) and WT vs *Rbm20*$^{S637A/S637A}$ (d).

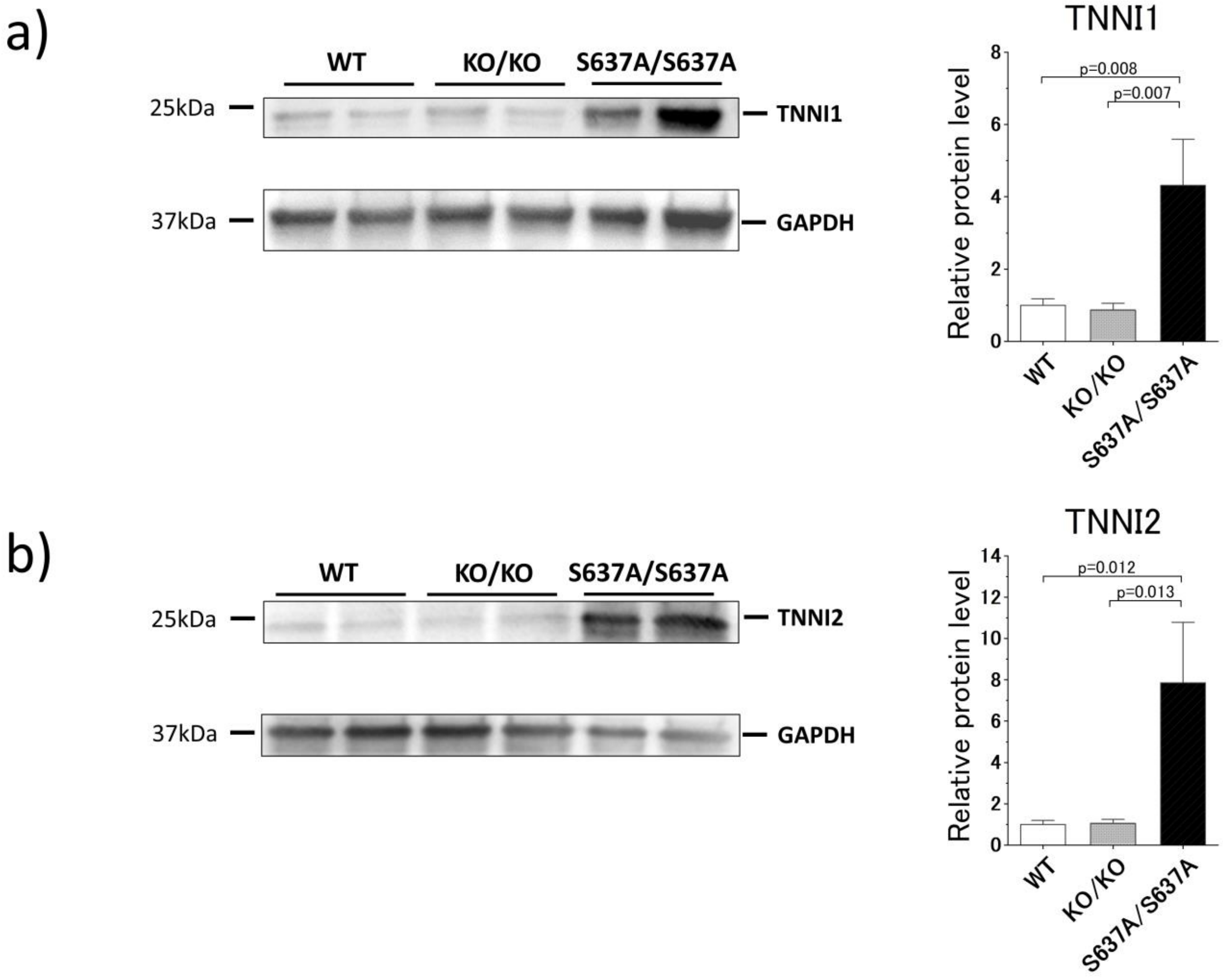

Figure S6. Western blot of TNNI1 (a) and TNNI2 (b) using proteins extracted from the hearts.

For quantitative analysis, n=3 each. P-values with statistical significance after Tukey's HSD test are indicated. Error bars, SEM. These results are consistent with those of RNA-seq (Fig. 6c) and qPCR (Fig. 6e).

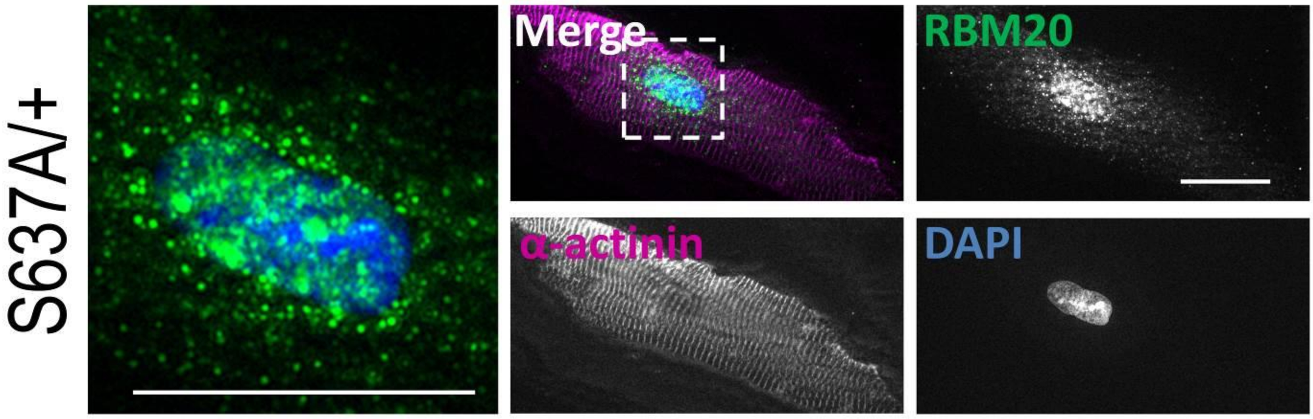

Figure S7. Immunofluorescence staining of *Rbm20*$^{S637A/+}$ cardiomyocytes reveals RBM20 in both the nuclei and the cytoplasm.

A dotted area is enlarged and shown on the left. Scale bars, 25 µm. n=3 mice, showing similar results.

Full-length gel-like image by Bioanalyzer for Figure 1a top panel. Cropped area is indicated.

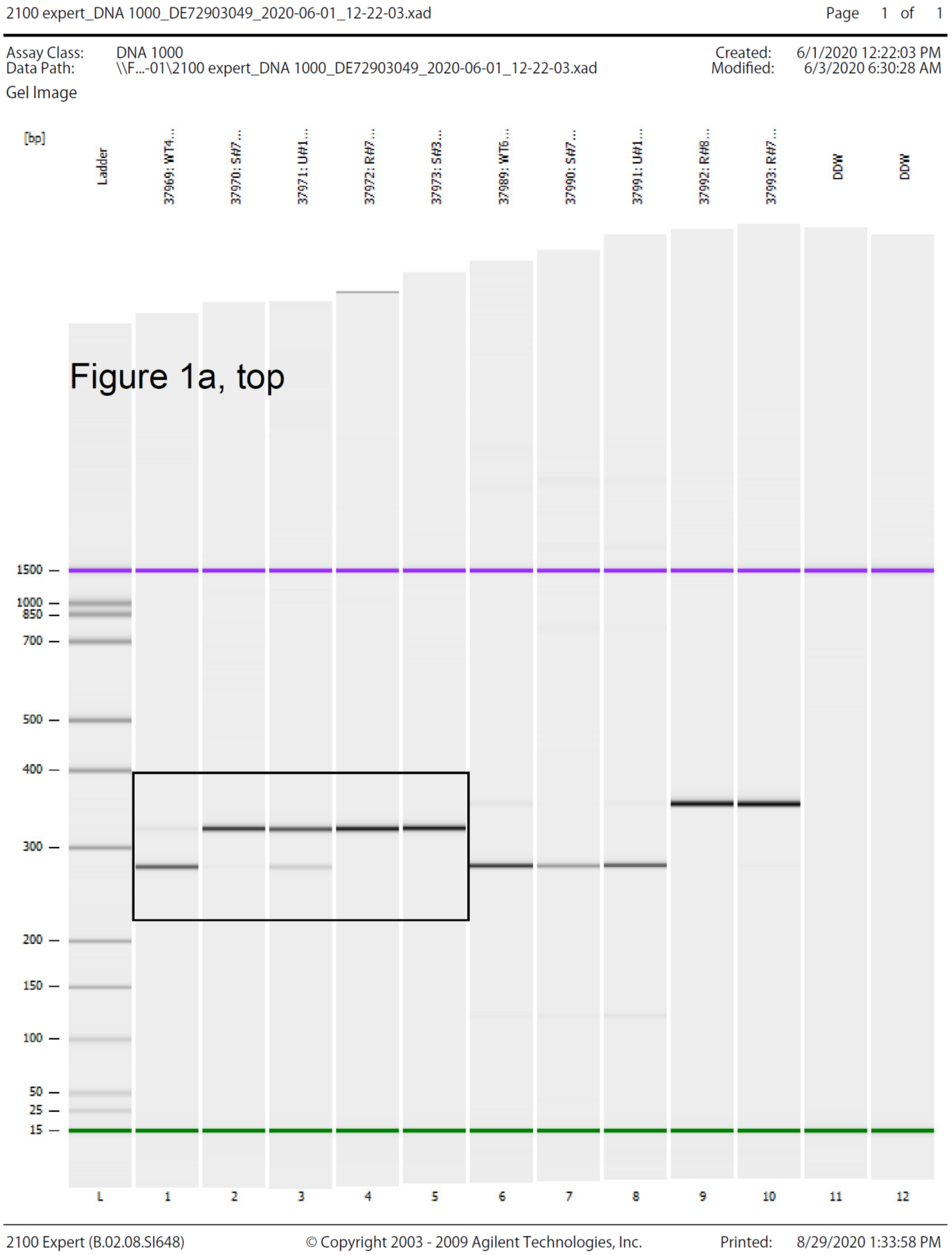

Full-length gel-like image by Bioanalyzer for Figure 1a middle and bottom panel. Cropped area is indicated.

2100 expert_DNA 1000_DE72903049_2020-06-01_11-03-59.xad Page 1 of 1

Assay Class: DNA 1000
Data Path: \\F...-01\2100 expert_DNA 1000_DE72903049_2020-06-01_11-03-59.xad

Created: 6/1/2020 11:03:59 AM
Modified: 6/3/2020 6:12:37 AM

Gel Image

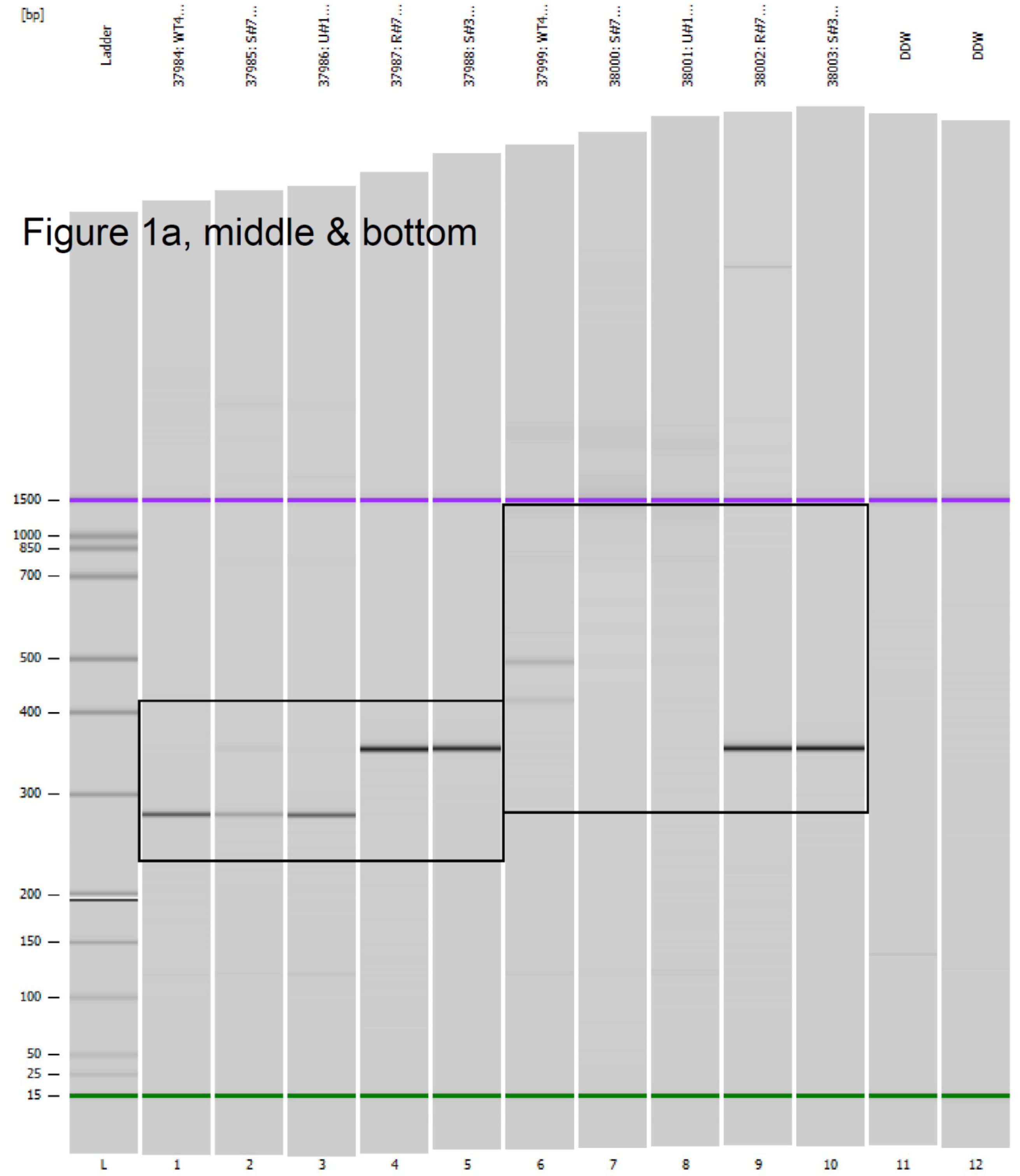

2100 Expert (B.02.08.SI648) © Copyright 2003 - 2009 Agilent Technologies, Inc. Printed: 8/29/2020 1:41:39 PM

Full-length gel-like image by Bioanalyzer for Figure 1b top and bottom panel. Cropped area is indicated.

Assay Class: DNA 1000
Data Path: \\F...-02\2100 expert_DNA 1000_DE72903049_2020-06-02_12-17-37.xad

Created: 6/2/2020 12:17:36 PM
Modified: 6/3/2020 7:23:10 AM

Gel Image

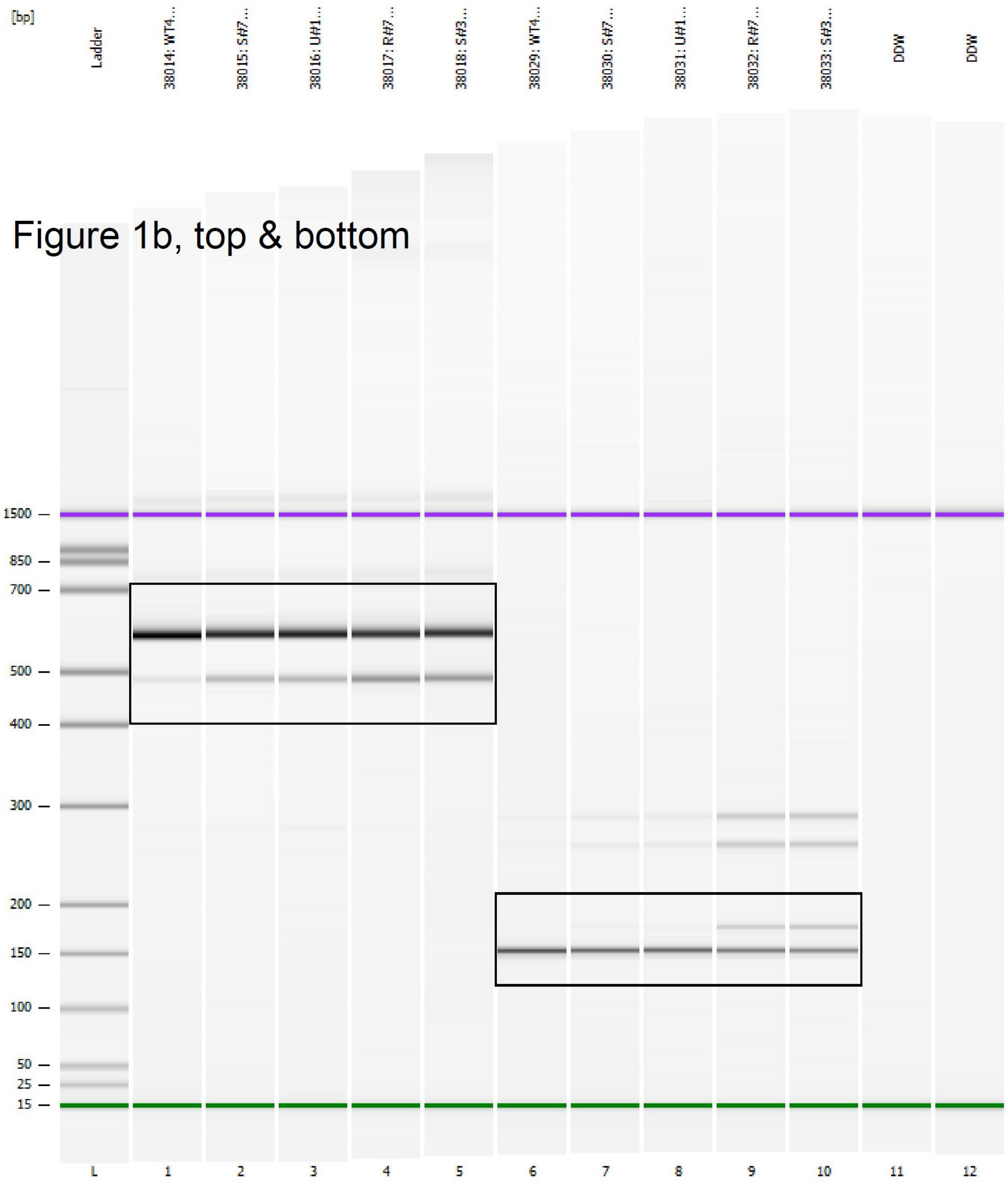

 © Copyright 2003 - 2009 Agilent Technologies, Inc.

Full-length gel-like image by Bioanalyzer for Figure 1b middle panel. Cropped area is indicated.

Assay Class: DNA 1000
Data Path: \\F...-02\2100 expert_DNA 1000_DE72903049_2020-06-02_13-52-44.xad
Created: 6/2/2020 1:52:44 PM
Modified: 6/3/2020 7:35:07 AM

Gel Image

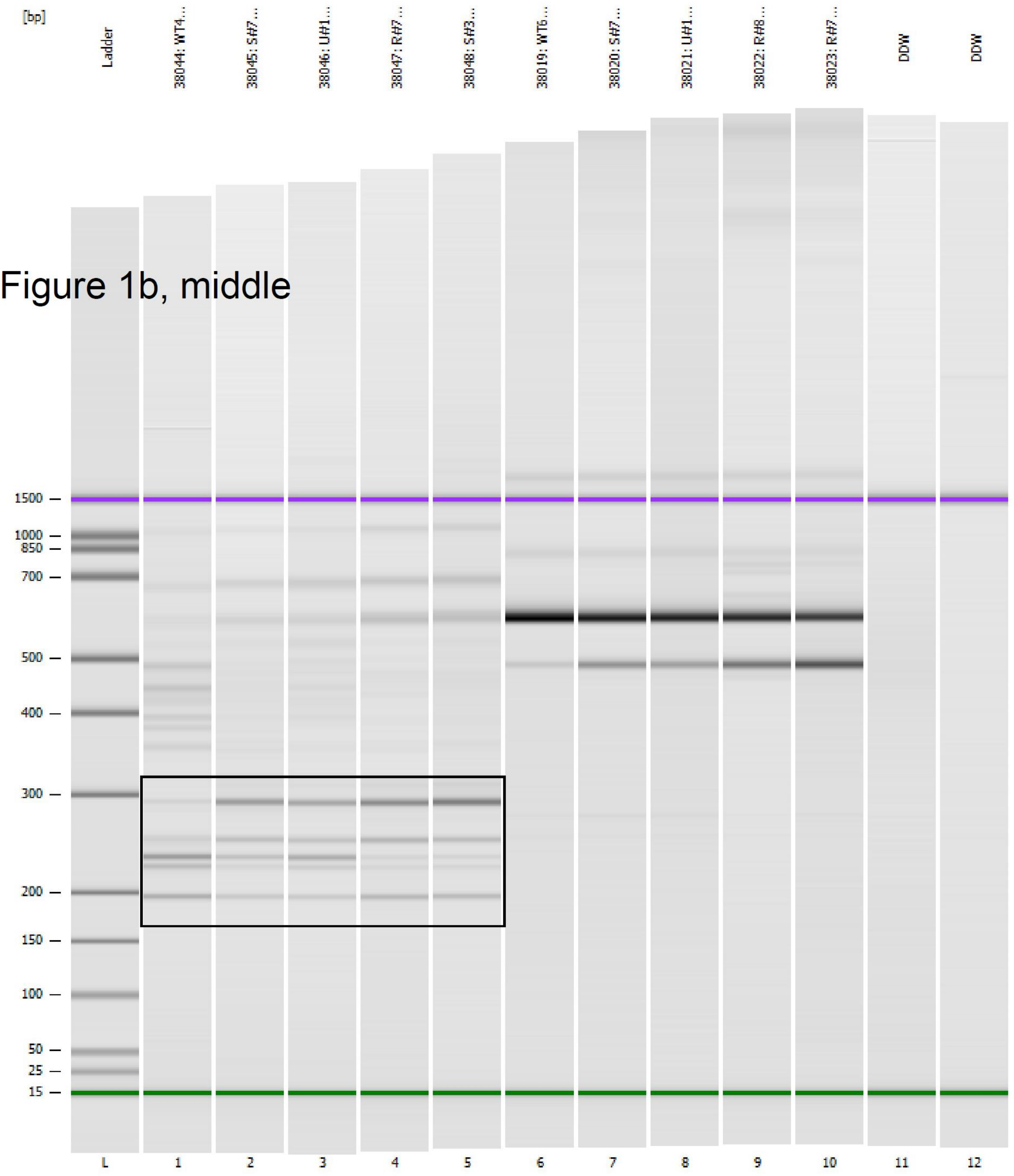

 © Copyright 2003 - 2009 Agilent Technologies, Inc.

Full-length images of gel for Figure 1c. Cropped area is indicated.

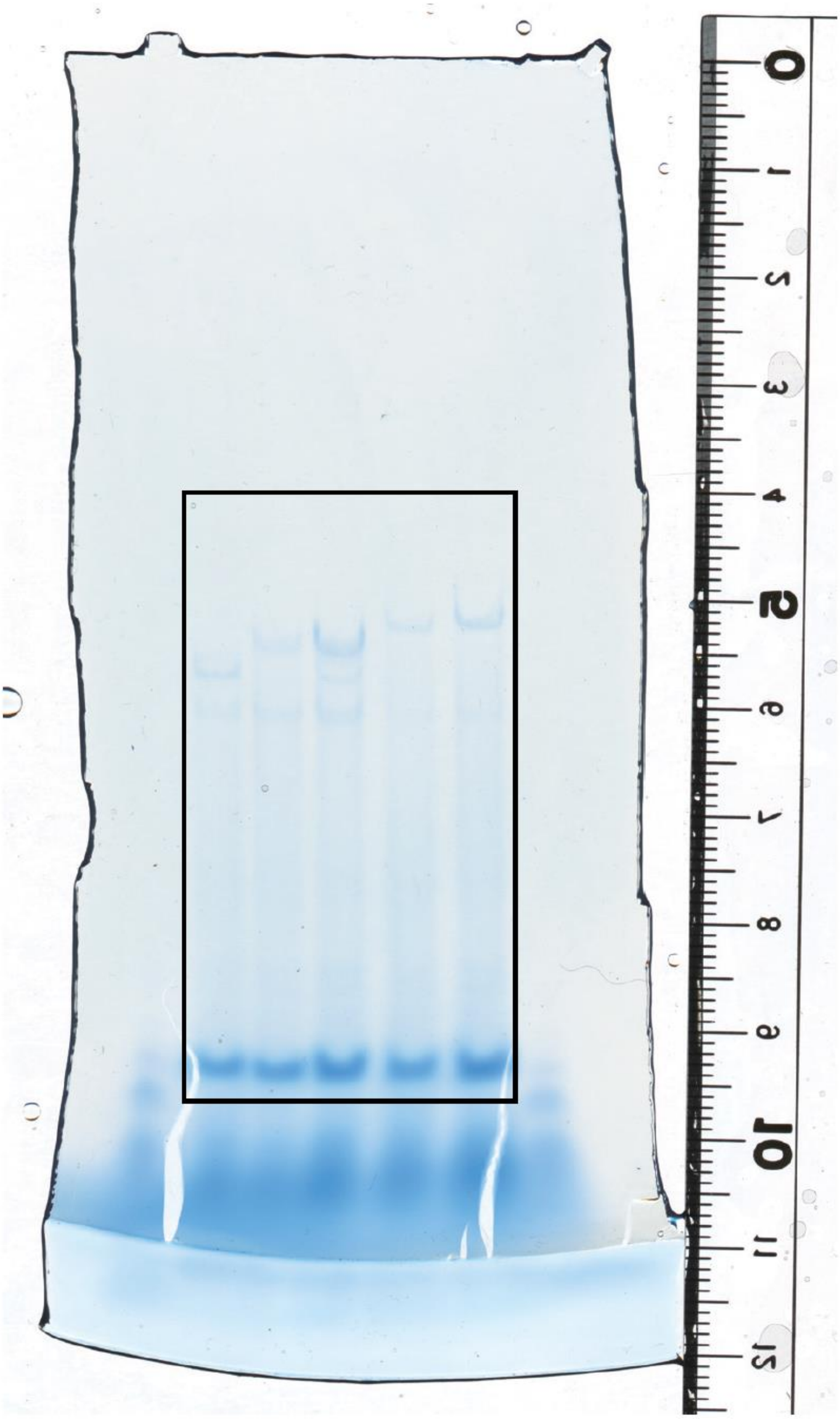

Full-length gel-like image by Bioanalyzer for Figure S1b. Cropped area is indicated.

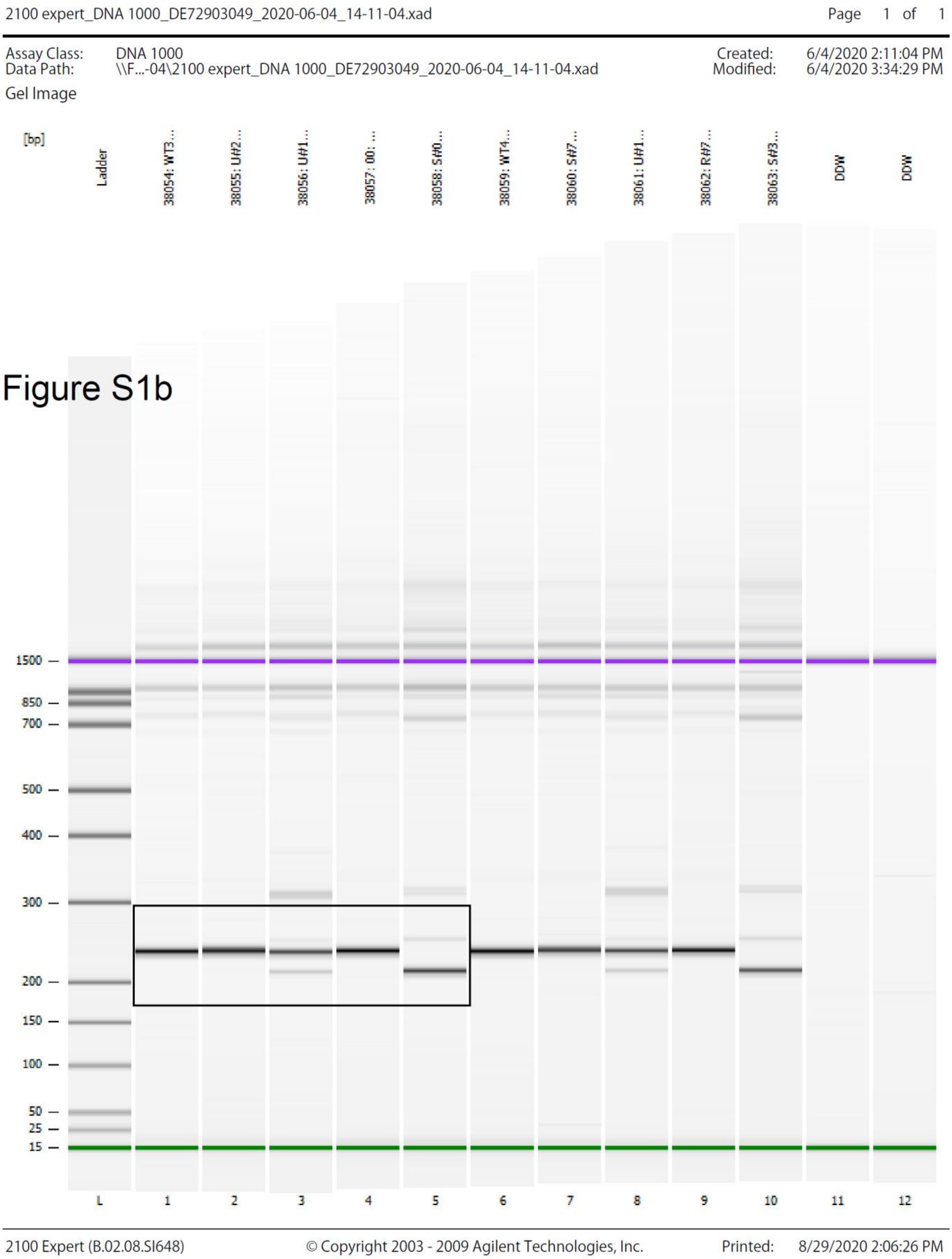

Full-length images of blot for Figure S1c. Cropped area is indicated.

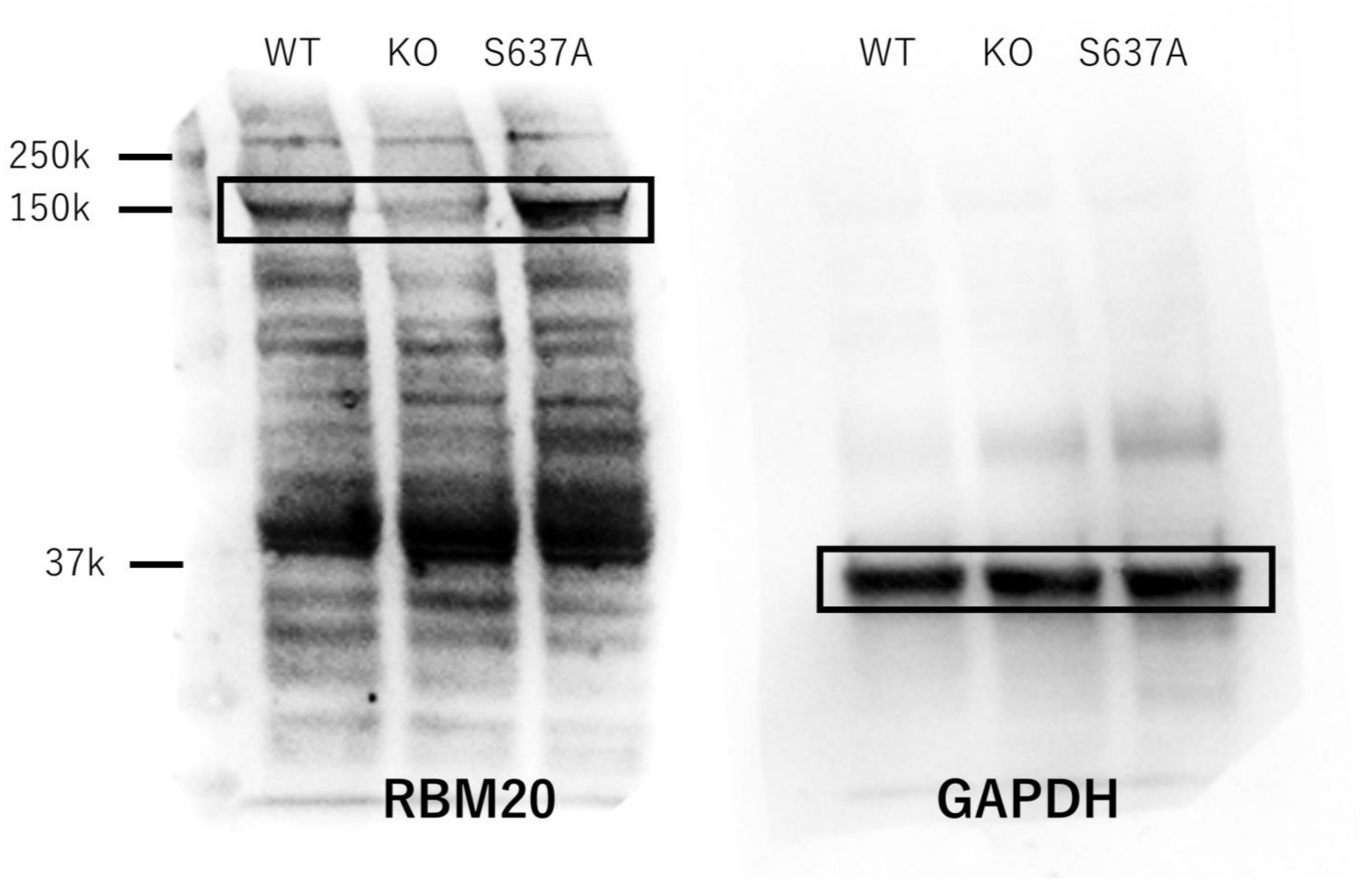

Full-length images of blot for Figure S6a. Cropped area is indicated.

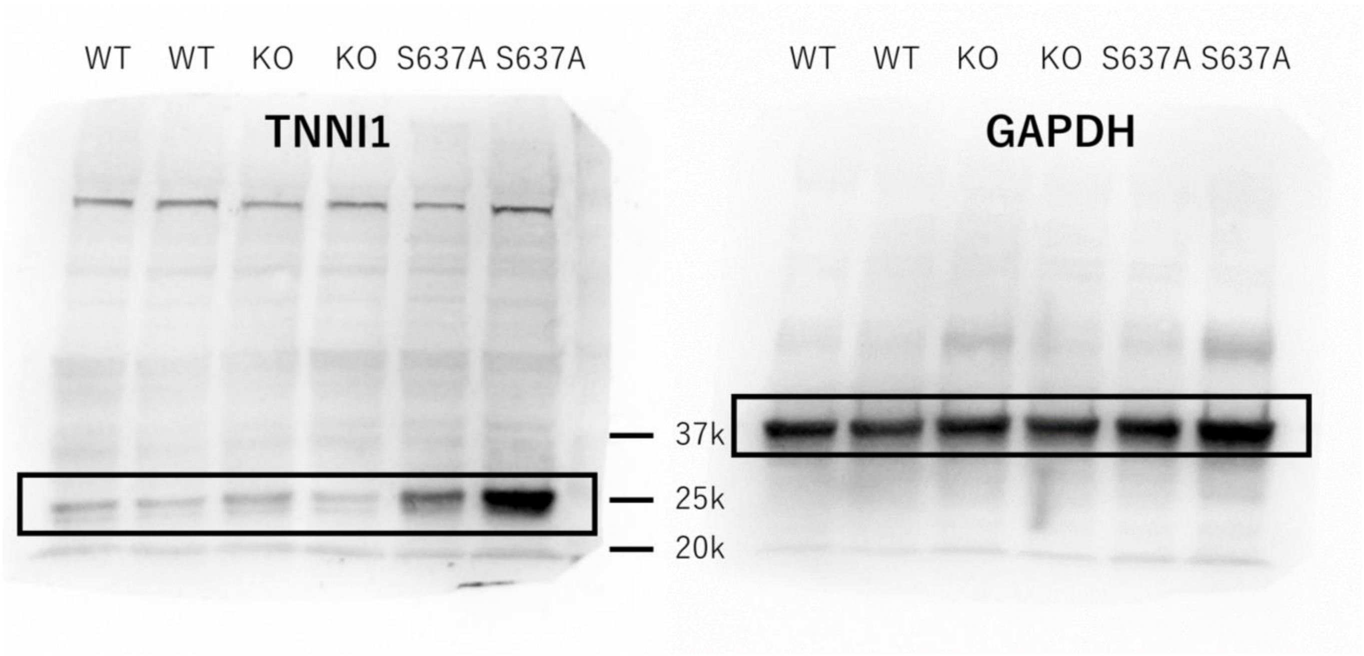

Full-length images of blot for Figure S6b. Cropped area is indicated.

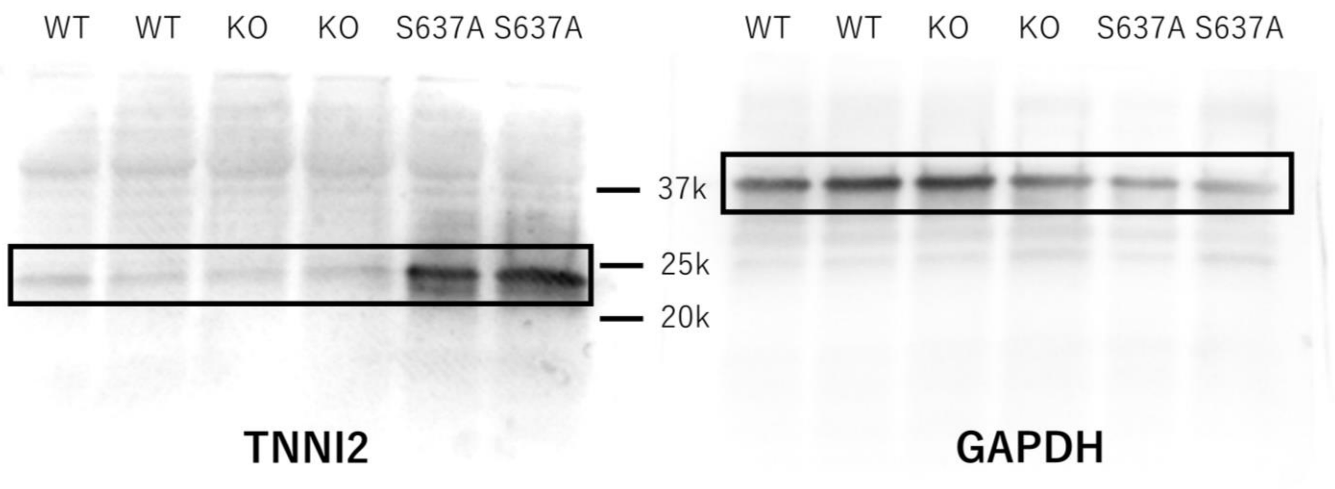

Supplement: Supplementary file 1 — Supplementary Information 1. [file 41598_2020_74800_MOESM1_ESM.pdf]
